# Regulating through-space charge transfer interactions in donor–acceptor MOFs for thermally activated delayed fluorescence and X-ray scintillators

**DOI:** 10.1039/d5sc02235e

**Published:** 2025-08-12

**Authors:** Xinyue Yan, Shi-Yu Song, Shicong Liang, Kai-Kai Liu, Xiao-Ting Liu, Chao Lu

**Affiliations:** a College of Chemistry, Pingyuan Laboratory, Zhengzhou University Zhengzhou 450001 China liuxiaoting@zzu.edu.cn luchao@mail.buct.edu.cn; b Henan Key Laboratory of Diamond Optoelectronic Material and Devices, Key Laboratory of Material Physics, Ministry of Education, School of Physics and Microelectronics, Zhengzhou University Zhengzhou 450001 China; c State Key Laboratory of Chemical Resource Engineering, Beijing University of Chemical Technology Beijing 100029 China

## Abstract

Through-space charge transfer (TSCT) thermally activated delayed fluorescence (TADF) materials have recently shown great potential for applications in X-ray detection and imaging due to their efficient triplet exciton utilization. By rational tuning of molecular/electronic structures of D and A moieties and precise regulation of TSCT interactions, tunable emission and TSCT-based TADF can be achieved. However, the TSCT interactions in organic D–A systems are somewhat difficult to design and control precisely resulting from relatively weak D–A supramolecular interactions. Herein, we propose a concept of TADF host–guest MOF scintillators with modulational TSCT interactions by confining D molecules as guests in the pore spaces of host MOFs composed of A ligands. Based on the synergy of the coordination and D–A interactions, various D molecules can be introduced into MOFs and manipulation of TSCT interactions can be achieved. As a result, tunable emission wavelengths spanning from 532 nm to 738 nm and successful construction of TADF host–guest MOF materials for X-ray scintillators can be achieved. Thanks to stable and enhanced triplet exciton utilization efficiency induced by TADF, the scintillator performance of compound 1 obviously surpassed that of separate D and A components as well as that of traditional organic commercial scintillator anthracene. The potential application of this scintillator in X-ray imaging was also demonstrated. This work provides a novel strategy for developing host–guest MOF scintillators.

## Abstract 2

Based on the synergy of coordination and D–A interactions, D guests have been introduced into MOFs composed of A ligands to manipulate TSCT interactions. As a result, TADF-type host–guest MOFs for X-ray scintillators have been achieved.

## Graphical abstract

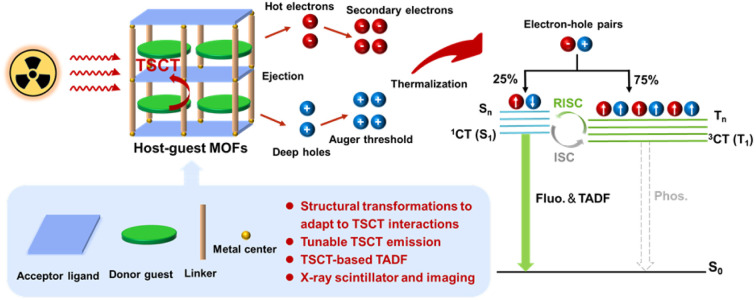

## Introduction

X-ray scintillators, which can convert high-energy X-rays into ultraviolet-visible photons, have attracted tremendous attention owing to their potential for diverse radiation applications.^1–8^ In particular, organic scintillators have become a research hotspot due to their intrinsic advantages of tunable photophysical properties and solution processing for devices.^9–11^ The organic molecules will be excited to both singlet and triplet states in the ratio of 1 : 3 by X-rays according to the spin conservation rule. However, only 25% of singlet excitons can be utilized by organic scintillators based on traditional fluorescent materials and 75% of triplet excitons will be wasted by the non-radiation processes.^12^ The low exciton utilization efficiency severely limits the development of organic fluorescent materials for X-ray scintillators. In recent years, thermally activated delayed fluorescence (TADF) organic materials have been reported to have been successfully used for X-ray scintillators, which can sufficiently exploit X-ray-induced triplet excitons through reverse intersystem crossing (RISC) from a triplet excited state (T_*n*_) to a singlet excited state (S_*n*_). In this sense, the theoretical exciton utilization rate can reach up to 100% for emission. This points to a new direction for development of high-performance scintillator materials.^13–15^

Through-space charge transfer (TSCT) organic molecules containing donor (D) and acceptor (A) moieties are an emerging class of emitters that can interact by weak electronic coupling through space.^16–20^ The character of effective frontier molecular orbital separation of TSCT organic molecules possesses the advantage of separately manipulating the molecular/electronic structures of D and A.^16,21,22^ This enables tuning the D–A interactions, and thereby exerting a significant influence on the TSCT states and TSCT-based singlet emission (emission wavelengths, PLQYs, lifetimes, *etc.*).^21^ More importantly, modulating various triplet state harvesting processes can also be achieved by the precise regulation of TSCT states.^23^ This is particularly important for achieving effective and modulable TADF by harvesting the triplet excitons to the singlet excited state. Therefore, TSCT has emerged as an appealing strategy to develop efficient TADF materials for X-ray scintillators. However, the TSCT interactions in organic D–A systems are somewhat difficult to design and control precisely resulting from uncontrollable assembly processes induced by the relatively weak D–A supramolecular interactions. Therefore, the quest for a highly flexible and controllable method to modulate TSCT interactions and obtain TADF-based scintillator materials remains challenging.

Metal–organic frameworks (MOFs) have emerged as advanced optical materials due to their diverse structures, customizable inorganic and organic components and highly tunable emissive properties.^24–29^ Aiming at the oriented construction and modulation of photophysical properties, we have developed a novel strategy *via* introducing the D and A moieties featuring TSCT interactions into MOFs.^30–32^ By confining D molecules as guests in the pore spaces of host MOFs composed of A ligands, the D-A host–guest MOF materials can be generated. The TSCT interactions can be modulated by rational regulation of the D and A species and structures of the host–guest MOFs, making D–A host–guest MOFs an ideal platform for manipulation and modulation of the excited states for tunable photophysical properties such as fluorescence and TADF. This approach presents a promising strategy for regulation of the TSCT interactions to design TADF materials for X-ray scintillators. Based on the synergy of the coordination between A ligands and metal centers and D–A interactions between A ligands and D guests, various D and A molecules can be introduced into the MOFs and the manipulation of the D–A arrangements can be achieved. As a result, the appropriate frontier molecular orbitals and matched spatial directions of the orbital electron cloud can be obtained.^33^ This facilitates the regulation of TSCT interactions and successful construction of TADF materials for X-ray scintillators. Additionally, flexible MOFs can allow diverse D guests into the host frameworks by adaptive structural transformations, increasing the modulability of TSCT interactions. Due to effective pore space filling, the obtained host–guest MOFs exhibit relatively dense D–A stacking structures and enhanced rigid character, which will contribute to substantially stabilizing the triplet excitons to avoid being quenched by oxygen and decreasing the non-radiative transition.^34,35^

**Scheme 1:**
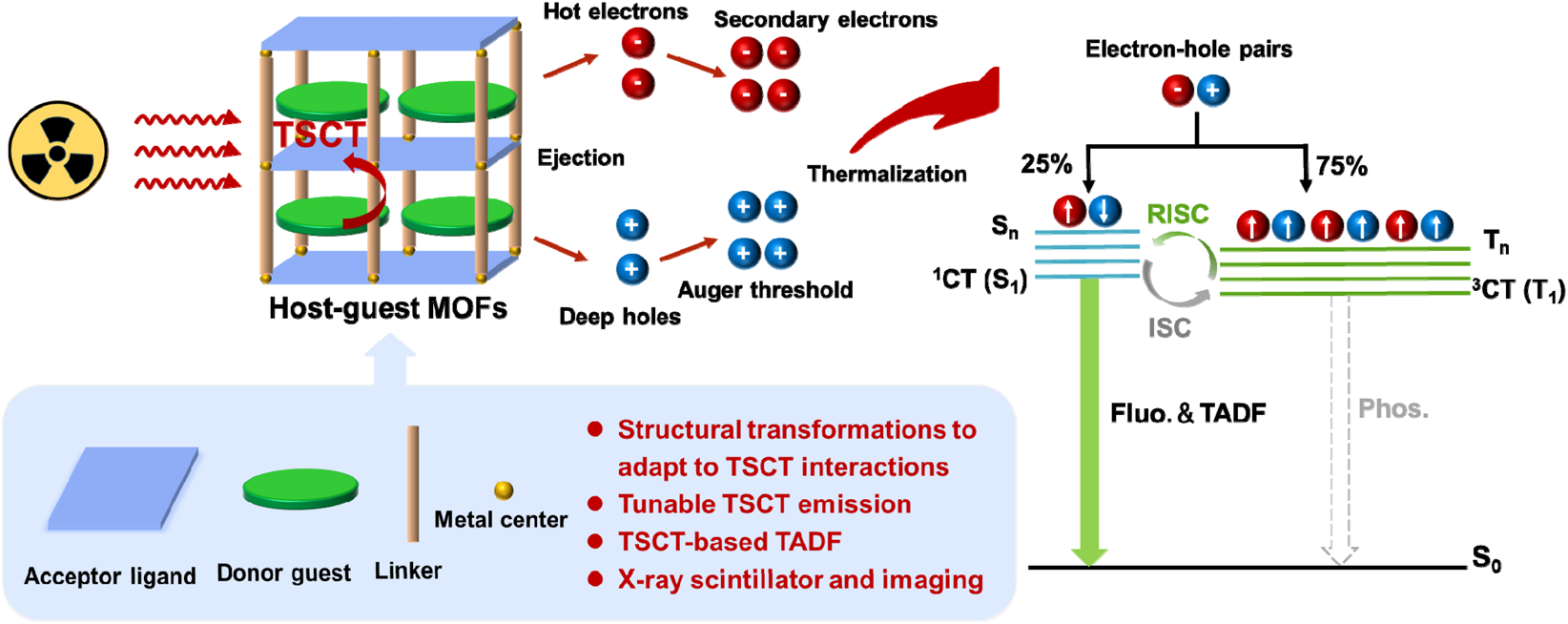
Mechanism illustration of TSCT-based TADF host–guest MOFs for the X-ray induced scintillator process.

With the above considerations in mind, we propose a concept of TADF host–guest MOF scintillators with modulational TSCT interactions for X-ray imaging applications (Scheme 1). To realize this concept, we synthesized three host–guest MOFs by employing Cd(ii) ions as the metal center, 2,4,6-tri(pyridin-4-yl)-1,3,5-triazine (tpt) as the A ligand, flexible biphenyl-4,4′-dicarboxylic acid (H_2_bpdc) as the auxiliary ligand and planar aromatic hydrocarbons (PAHs, triphenylene for compound 1, perylene for compound 2, and coronene for compound 3) as the D guests. These three MOFs exhibited distinct structures owing to the need to match the D–A interactions by an adaptive structural change. Resulting from different TSCT interactions induced by D species, tunable emission wavelengths spanning from 532 nm to 738 nm were achieved. Meanwhile, precise regulation of the TSCT state between tpt and triphenylene in compound 1 resulted in TSCT-based TADF properties. The X-ray scintillating properties of compound 1 were further studied. The radioluminescence (RL) intensity of 1 obviously surpassed that of the separate D and A components as well as traditional organic commercial scintillator anthracene. Besides, the RL intensities of compound 1 showed a good linear response to the dose rates of X-rays, revealing a detection limit of 1570 nGy_air_ s^−1^, surpassing the medical diagnostic standard of 5.5 μGy_air_ s^−1^. Compound 1 also achieved a resolution of 10.7 lp mm^−1^. The superior performance enabled high-resolution X-ray imaging. A Polydimethylsiloxane (PDMS) film with powder of compound 1 was fabricated to perform X-ray radiography, highlighting promising applications in bioimaging, medical diagnosis, *etc.* This work provides a novel strategy for developing host–guest MOF scintillators and may open the way for advancements in MOF scintillator research.

## Results and discussion

### Construction and characterization of host–guest MOFs

**Fig. 1:**
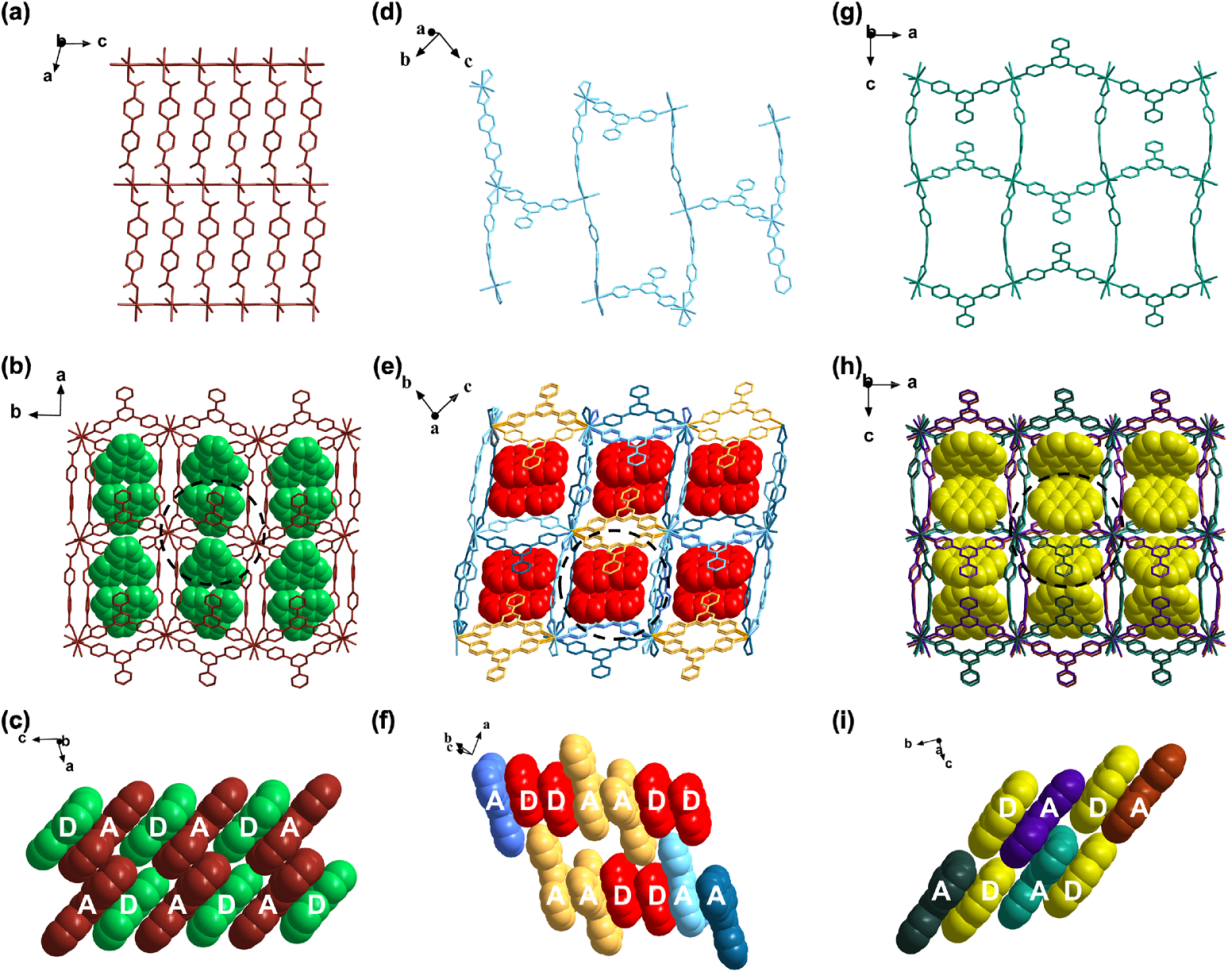
The structure of compound 1: (a) the 2D layer viewed along the *b*-axis; (b) the 3D host–guest network viewed along the *c*-axis; (c) the stacking of triphenylene (D) and tpt (A). The structure of compound 2: (d) the 2D layer viewed along the *a*-axis; (e) the 3D host–guest network viewed along the *a*-axis; (f) the stacking of perylene (D) and tpt (A). The structure of compound 3: (g) the 2D monolayer viewed along the *b*-axis; (h) the 2D host–guest network viewed along the *b*-axis; (i) the stacking of coronene (D) and tpt (A).

Based on our previous research on D–A host–guest MOFs,^30–32^ 2,4,6-tri(pyridin-4-yl)-1,3,5-triazine (tpt) as the A ligand, flexible biphenyl-4,4′-dicarboxylic acid (H_2_bpdc) as the auxiliary ligand, Cd(ii) ions as the metal centers and planar aromatic hydrocarbons (PAHs) as the D guests were utilized for the construction of host–guest MOFs. The solvothermal reactions of these components in a mixture of DMF/EtOH/H_2_O resulted in high-quality bulk crystals of 1–3 (triphenylene in compound 1, perylene in compound 2, and coronene in compound 3). SCXRD analysis reveals that compound 1 crystallizes in the *P*2_1_/*c* space group of the monoclinic system. The asymmetric unit contains one Cd(ii) ion, one tpt ligand, one linear bpdc^2−^ ligand, one coordinated H_2_O molecule and one free triphenylene molecule (Fig. S1). The Cd(ii) ion adopts a twisted [N_2_O_4_] octahedral geometry by coordinating with four oxygen atoms from two bpdc^2−^ ligands, two H_2_O molecules, and two pyridyl nitrogen atoms from two tpt ligands. Each bpdc^2−^ ligand adopts mono-bridging mode to link two Cd(ii) ions to form the one-dimensional (1D) chains (Fig. S4). The nearest distance of Cd(ii) centers in adjacent 1D chains is 4.637 Å, and the 1D chains can be extended to two-dimensional (2D) layers by linking coordinated H_2_O molecules and different Cd(ii) centers in adjacent chains (Fig. 1a). The 2D layers can further be connected by two pyridyl nitrogen atoms from each tpt ligand to generate an extended three-dimensional (3D) framework, in which the pore spaces are occupied by triphenylene molecules (Fig. 1b). In the whole framework, the tpt and triphenylene exhibit a face-to-face dense DADADA stacking mode viewed along the *b*-axis (Fig. 1c and S5). The effective vertical distance of tpt and triphenylene is 3.2733 Å (Fig. S6). This guarantees that the D and A are completely spatially separated and arranged in proximity for the occurrence of TSCT interactions.^36^

By increasing the dimension of the guest by replacing triphenylene with perylene, compound 2 can be obtained. Compound 2 crystallizes in the triclinic system with the *P*1̄ space group and consists of two Cd(ii) ions, two tpt ligands, two linear bpdc^2−^ ligands, two coordinated H_2_O molecules and two free perylene molecules in the asymmetric unit (Fig. S2). The two Cd(ii) ions are coordinated by the same [N_2_O_5_] pentagonal bipyramidal geometry defined by one oxygen atom from one coordinated H_2_O, four oxygen atoms from two bpdc^2−^ ligands and two pyridinyl nitrogen atoms from two tpt ligands. Each bpdc^2−^ ligand adopts a bidentate chelating mode to link two Cd(ii) ions to adapt to the increased D–A interactions induced by the larger dimension of the perylene (Fig. S4), and 1D chains can be generated. The conformations of the bpdc^2−^ ligands become twisted as well. Accordingly, the nearest distance of Cd(ii) centers in adjacent 1D chains increases (5.605 Å), and so Cd(ii) centers in adjacent 1D chains can't be linked by coordinated H_2_O molecules. There are two kinds of tpt ligands with the same coordination modes but different conformations. The 1D chains are connected by one kind of tpt ligand to generate 2D layers (Fig. 1d), and these 2D layers are further linked by the other kind of tpt ligand to generate a 3D framework (Fig. 1e). The pore spaces of the 3D framework are occupied by perylene molecules and tpt and perylene exhibit a face-to-face dense DDADDA stacking mode viewed along the *c*-axis (Fig. 1f and S5). The effective vertical distance of tpt and perylene is 3.3864 Å (Fig. S6).

Upon further increasing the dimension of the guest by replacing triphenylene with coronene to increase the D–A interaction, compound 3 was obtained. Compound 3 crystallizes in the *Cmc*2_1_ space group of the orthorhombic system. The asymmetric unit contains one Cd(ii) ion, one tpt ligand, one linear bpdc^2−^ ligand, one coordinated H_2_O and one coronene molecule (Fig. S3). The Cd(ii) ion is coordinated by one oxygen atom from one H_2_O molecule, four carboxyl oxygen atoms from two bpdc^2−^ ligands, and two pyridinyl nitrogen atoms from two tpt ligands, forming a [N_2_O_5_] pentagonal bipyramidal geometry. Similarly, each bpdc^2−^ ligand adopts a bidentate chelating mode to link two Cd(ii) ions to adapt to the increased D–A interaction and the nearest distance of Cd(ii) centers in adjacent 1D chains is 5.514 Å (Fig. S4), and so Cd(ii) centers in adjacent 1D chains can't be linked by coordinated H_2_O molecules. Instead, the 1D chains are connected by tpt ligands to generate a 2D network, in which the pore spaces are occupied by coronene molecules (Fig. 1g and h). Although the tpt and coronene exhibit a face-to-face dense DADADA stacking mode viewed along the *a*-axis, which is similar to that in compound 1, the whole stacking of D and A molecules exhibits a zigzag mode owing to the bigger steric effect induced by coronene molecules (Fig. 1i and S5). The effective vertical distance of D and A is 3.5048 Å (Fig. S6). These results suggest that the introduction of flexible auxiliary ligands contributes to generating diverse host–guest MOFs by structural transformation to adapt to distinct D–A interactions. In this sense, host–guest MOFs can be a suitable platform for manipulation of D–A spatial alignments to regulate the TSCT interactions for TADF and TADF-based X-ray scintillators.

The successful encapsulation of the guests was further verified by the digested ^1^H NMR and elemental analysis, suggesting a full loading of all the pore voids of compounds 1–3 (Fig. S7–S9). The phase purity and stability of the as-synthesized materials were validated by powder X-ray diffraction (PXRD) and thermogravimetric (TG) analysis, indicating high phase purity and good thermal stability up to 400 °C (Fig. S10–S13).

### Tunable TSCT interactions in host–guest MOFs for TADF

Usually, the CT character can be regulated by introducing varied D and A with different energy levels.^37^ For instance, the intensified CT character can be achieved by introducing strong D or A rather than weak D or A. Generally, the strong donors will afford high HOMOs and strong acceptors will provide low LUMOs. Therefore, by the rational choice of varied D with different HOMOs and A with different LUMOs, the CT interactions can be tuned. In our three host–guest compounds with the TSCT character, the HOMOs are located on the D (PAH) guests and the LUMO is located on the A (tpt) ligand. Based on this, we first calculated the orbital energy levels of the tpt ligand and the three PAH guests (Fig. S14). For the tpt acceptor with the same LUMO, the high HOMOs of PAH guests (*E*_(HOMO)_(perylene) > E_(HOMO)_(coronene) > *E*_(HOMO)_ (triphenylene)) afford strong electron-donating ability, which may contribute to intensifying the TSCT character.

However, besides the energy levels of D and A molecules induced by varied molecular/electronic structures of D and A that can affect the TSCT interactions, the relative positions between the tpt and the corresponding guests could also influence the TSCT interactions. Detailed structural analysis reveals that the relative positions between tpt and corresponding guests were distinct in compounds 1–3 owing to the relatively weak confinement effect of the host pore spaces induced by adaptively structural transformations to match the D–A interactions. This could influence the overlap degrees of π systems and TSCT interactions. The more effective overlap of the tpt and guest molecules will result in more effective charge transfer.^38,39^ The relative positions can be related to the vertical distances (a) and the areas of face–face stacking (b) between A (tpt ligand) and D (PAH guests) in compounds 1–3 (Fig. S6). Based on this, a simplified model for comparing relative positions between the A ligand and D guests was constructed, of which *c* that represented the distances between center A and center D, could reflect the relative positions. The values of *c* were 3.5240 Å in 1, 3.6727 Å in 2 and 3.6847 Å in 3. Considering the similar *c* values in compounds 2 and 3, the higher HOMO of perylene in 2 than that of coronene in 3 could increase TSCT interaction. Although the *c* value in compound 1 was the smallest compared to those in compounds 2–3, the relatively low HOMO of triphenylene in 1 was not conducive to the TSCT interaction. Overall, besides the energy levels of D and A that can affect the TSCT interactions, the relative positions of D and A should also be considered for tuning TSCT interactions and TSCT originated properties.

**Fig. 2:**
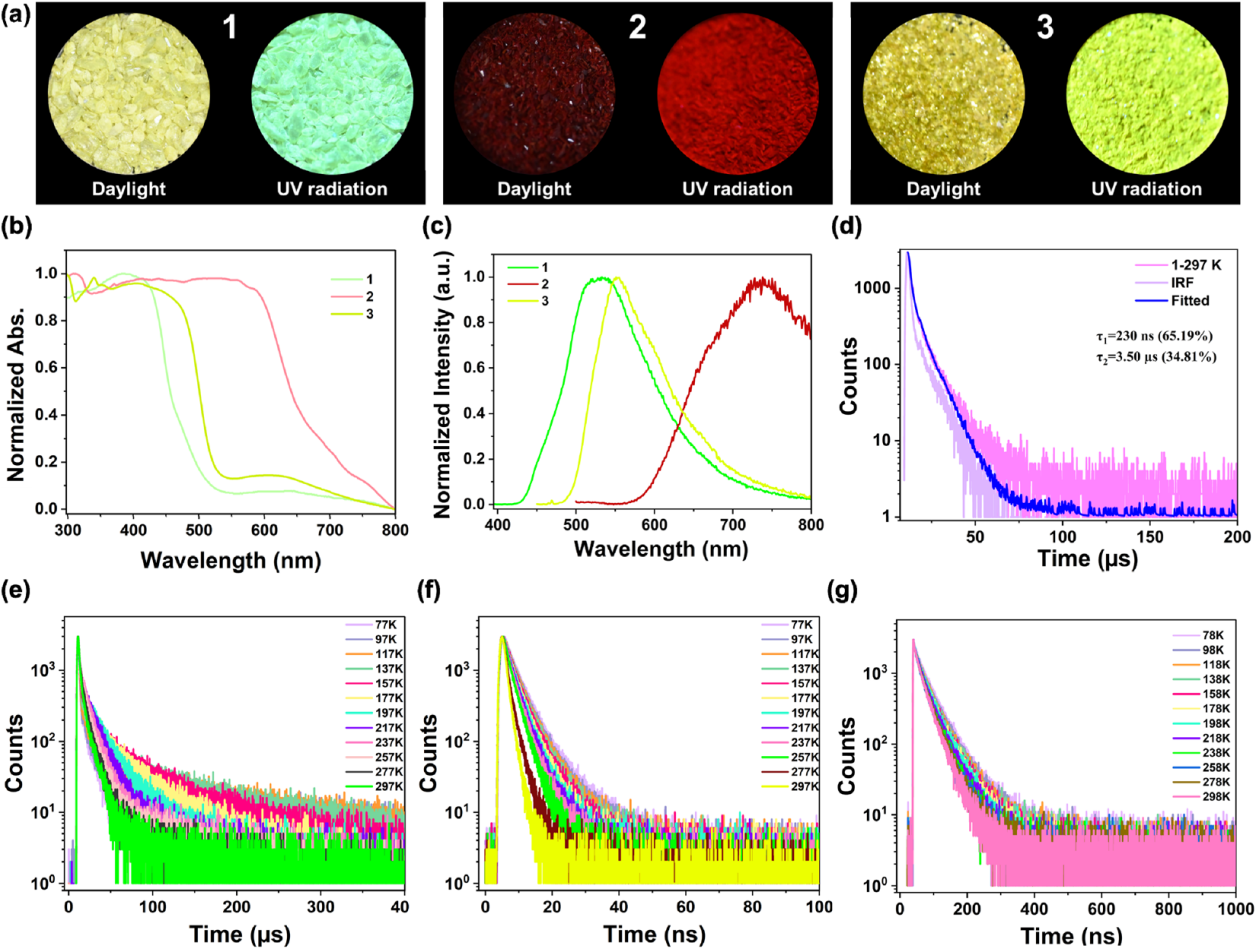
(a) The photographs of crystalline samples of 1, 2 and 3 under daylight and UV radiation; (b) and (c) UV-vis absorption spectra and PL spectra of compounds 1, 2 and 3; (d) the transient PL decay curve of compound 1 at 532 nm; (e) the temperature-dependent PL decay curves of compound 1 at 532 nm; (f) the temperature-dependent PL decay curves of compound 2 at 738 nm; (g) the temperature-dependent PL decay curves of compound 3 at 553 nm.

Based on the above analysis, solid-state ultraviolet-visible (UV-vis) absorption spectra of compounds 1–3 were recorded to verify the TSCT interactions. As shown in Fig. S15–S17, these three host–guest MOFs displayed new intense absorption bands in the long-wavelength regions compared with the tpt ligand and corresponding guest molecules, which were consistent with their visible colors (Fig. 2a). This can be attributed to the occurrence of TSCT interactions between the tpt ligand and guests.^40^ More detailed calculations were performed to obtain deeper insights into TSCT interactions and the details are shown in the Computational study section (Fig. S26–S31 and 3). The electron transition density analysis of the excited state at ground state geometries, which reflected the absorption, revealed that the holes were located on the PAH donors and the electrons were located on the tpt acceptor, which affirmed the TSCT interactions in the three compounds. The proportions of CT states of the lowest two singlets (S_1_, S_2_) computed at ground state geometries for 1 were 95%, 95%, for 2 were 98%, 98%, and for 3 were 93%, 93%, respectively (Table S2). Such high proportions revealed sufficient TSCT transitions. The computed vertical excitation energy gaps for S_1_/S_2_ in 1 were 3.165/3.172 eV with oscillator strengths, *f* = 4.60 × 10^−9^ and 0.0102, respectively. The computed vertical excitation energy gaps for S_1_/S_2_ in 2 were 2.282/2.283 eV with oscillator strengths, *f* = 0.0035 and 2.85 × 10^−7^, respectively. The computed vertical excitation energy gaps for S_1_/S_2_ in 3 were 2.885/2.885 eV with oscillator strengths, *f* = 0.0002 and 8.13 × 10^−8^, respectively. Considering that transitions with oscillator strength too small (<0.0001) can be approximately regarded as forbidden transitions, the effectively computed vertical excitation energy gaps were 3.172 eV for 1, 2.282 eV for 2 and 2.885 eV for 3, respectively (Table S3). For compounds 1, 3 and 2, the ranges of new absorption bands were at around 400–500 nm for 1, 450–500 nm for 3 and 500–650 nm for 2 (Fig. 2b). This significantly red-shifted tendency in the new absorption bands, which was consistent with the variation tendency of the vertical excitation energy gap, suggests gradually increased TSCT interactions from 1, 3 to 2.^41^

**Fig. 3:**
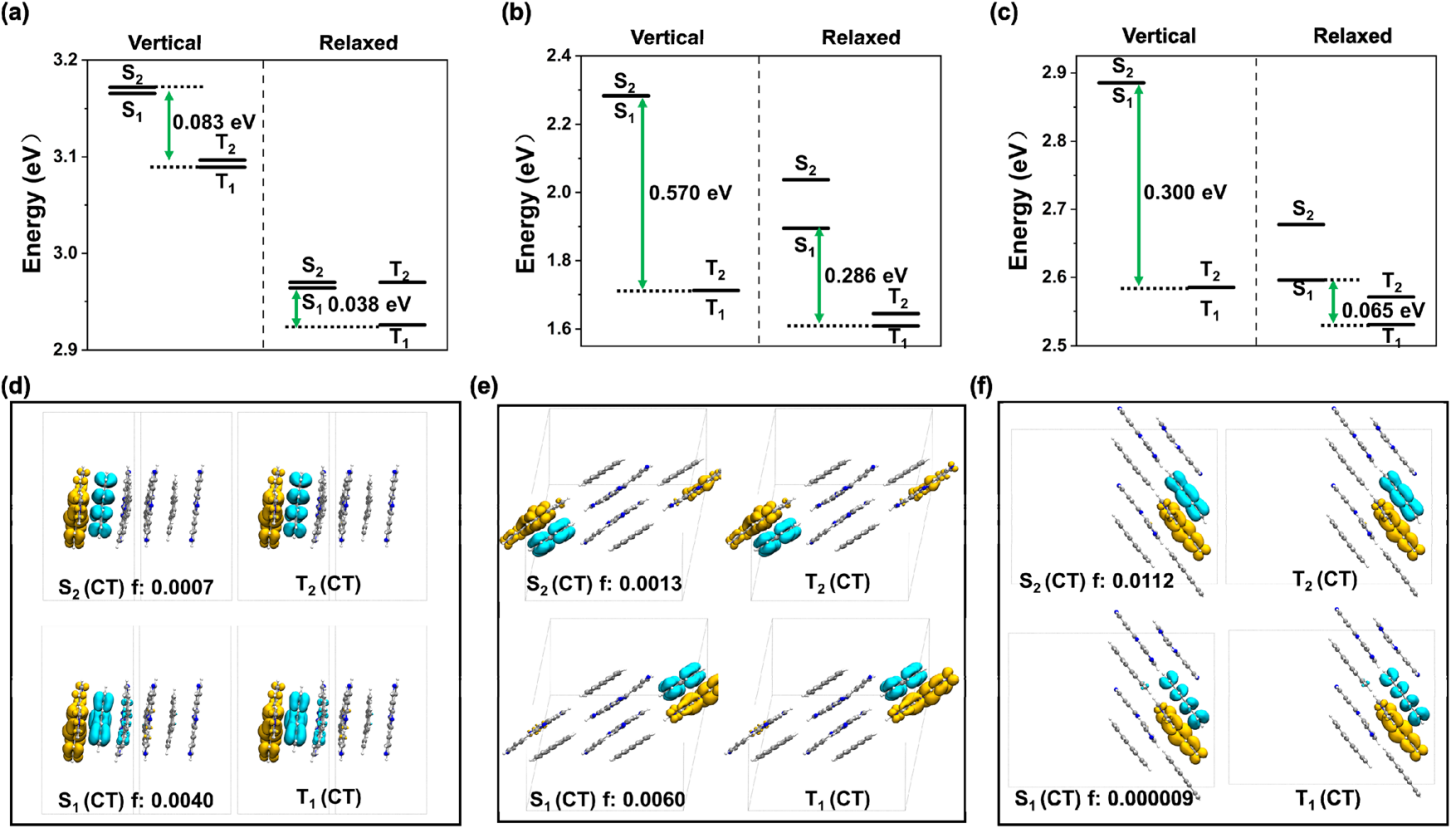
(a–c) Computed vertical excitation energy levels and relaxed excitation energy levels of compounds 1–3; (d)–(f) electron density transition analysis for the two lowest singlet/triplet states at optimized excited state geometries for compounds 1–3, and all were characterized as CT states (blue, hole; yellow, electron; isovalue: 0.0004).

The photoluminescence (PL) spectra of compounds 1–3 were also recorded. As shown in Fig. S18–S20, the PL spectra of compounds 1–3 exhibited broad, featureless and red-shifted emission bands with peaks at around 532 nm, 738 nm and 553 nm compared with their corresponding guests and tpt ligand, which revealed a typical TSCT nature. The electron transition density analysis of the excited states at the optimized excited state geometries (relaxed excitation), which corresponded to the PL emission, also revealed that the holes were located on the PAH donors and the electrons were located on the tpt acceptor, which reaffirmed the TSCT interactions in the three compounds. The effectively relaxed excitation energy gaps were 2.964 eV for 1, 1.895 eV for 2 and 2.596 eV for 3, respectively (Table S3). The variation tendency of the relaxed excitation energy gaps was consistent with red-shifted degrees of emission wavelengths (Fig. 2a and c), which can also reveal an increased TSCT interaction from 1, 3, to 2.^37,42^

In addition, the photoluminescence quantum yields (PLQYs) of compounds 1–3 were measured by the integrating sphere method at room temperature, which were 9.3%, <1% and 2.5%, respectively (Table S4). The relatively higher PLQY value of compound 1 can be assigned to the efficient radiative transition originating from relatively modest TSCT interaction to generate the CT excited state. However, the strong TSCT interactions in 2 and 3 facilitated the conversion of CT states to radical-ion pairs in the excited states, thus quenching the emission of 2 and 3 accordingly.^43^ These results suggest that the relatively modest TSCT interactions in D–A MOFs could suppress exciton dissipation caused by non-radiative transitions, which lays the foundation for achieving efficient RISC for TADF emission.

To investigate the TSCT interactions in modulating various excited-state processes in detail, the transient PL decay curves of compounds 1–3 were measured at room temperature at 532 nm, 738 nm and 553 nm, respectively (Fig. S21, S22 and 2d). The PL decay profile of 1 exhibited a nanosecond-scale prompt component with a lifetime (*τ*) of 230 ns and a microsecond-scale delayed component with *τ* = 3.50 μs while that of 2 was only one nanosecond-scale prompt component (the *τ* value is 1.24 ns). The PL decay profile of 3 exhibited two nanosecond-scale prompt components (the *τ* values are 12.48 ns and 34.75 ns), which were related to the deactivation of different excited states, and have been reported in other D–A type materials^43,44^ This result suggests that a triplet-harvesting channel is only available in compound 1, which involves the RISC/ISC processes between T_*n*_ and S_*n*_. To verify the TADF or phosphorescence process in compound 1, temperature-dependent PL spectra of 1 were recorded from 77 K to 297 K with an interval of 20 K. As shown in Fig. S23, upon increasing the temperature, the PL intensity increased first and then remained almost unchanged until 237 K. From 237 K to 297 K, the intensity exhibited a decreasing tendency, and also, the emission intensity at 297 K was still higher than that in 77 K. This is obviously different from fluorescence and phosphorescence, which commonly demonstrate a decreasing tendency of the emission intensity upon heating. The multiple peaks at low temperatures originated from the coupling of molecular vibrations. Variable-temperature PXRD indicated the integrity of the framework and non-deformable nature due to the lack of a shift or the absence of diffraction peaks (Fig. S24). Subsequently, the temperature-dependent PL decay curves of 1 were obtained from 77 K to 297 K (Fig. 2e and Table S5). The proportions of delayed components corresponding to the microsecond-scale lifetimes increased upon increasing the temperature from 77 K to 237 K, indicating a thermally activated PL process from the RISC process from T_*n*_ to S_*n*_, which is typical of TADF materials.^44,45^ Upon further increasing the temperature, the proportions of delayed components reduced slightly, which may originate from the dissipation of triplet excitons through nonradiative transitions caused by the thermal effect, which is in accordance with the decreased PL emission intensity from 237 K to 297 K. Meanwhile, the temperature-dependent PL decay curves of compounds 2–3 were also recorded from 77 K to 297 K (Fig. 2f, g, Tables S6 and S7). They all exhibited nanosecond-scale components and the average lifetime values decreased with increasing temperature, typical of fluorescence character.

To determine the Δ*E*_ST_ of compound 1, which is a critical factor for verifying TADF, the steady-state PL spectrum at 297 K and delayed PL spectrum at 77 K were measured and compared (Fig. S25). Singlet energy was estimated from the onset wavelength of the PL spectrum at 297 K (430 nm), and triplet energy was estimated from the onset wavelength of the delayed PL spectrum at 77 K (450 nm).^46^ From the equation (Δ*E*_ST_ (eV) = 1240/430–1240/450), the Δ*E*_ST_ was estimated to be 0.13 eV, which is less than 0.2 eV required for conventional TADF materials. These results reveal that rational TSCT interactions contribute to the excited-state process modulation and thus triplet state harvesting can be achieved.

To further understand the structures and photophysical mechanisms of compounds 1–3, the theoretical calculations were performed with three-dimensional simplified models of 1, 2 and 3 extracted from crystal structures with periodically stacked guests and ligands (Fig. 3 and S26–S31). The electron transition density analysis to the lowest excited-states (S_1_, S_2_, T_1_, and T_2_) computed at both ground and optimized excited state geometries revealed that all three compounds exhibited TSCT properties, in which the holes were located on the PAH donors and the electrons were located on the tpt acceptor. This means that the charge (electron) transfer occurred from PAHs to the tpt ligand in the three compounds. Considering the oscillator strength values, the effective Δ*E*_ST_ values for compounds 1–3 were calculated to be 0.083 eV, 0.570 eV and 0.300 eV, respectively, based on computed vertical excitation energy levels. The Δ*E*_ST_ values of compounds 1–3 were calculated to be 0.038 eV, 0.286 eV and 0.065 eV, respectively, based on computed relaxed excitation energy levels (Table S3). Evidently, despite different calculation methods, the Δ*E*_ST_ of compound 1 is always sufficiently small, which facilitates the RISC channel for effective TADF.

Furthermore, the photophysical properties of the physical mixture of triphenylene (D) and tpt (A) (molar ratio 1 : 1, according to the formula of compound 1), as a control, were compared. The steady-state PL spectrum at 297 K showed a series of strong peaks in the range of 350–450 nm and weak emission peaks at longer wavelengths (*ca.* 550–700 nm), which were assigned to the mixture of tpt and triphenylene (Fig. S32). The distinct emission spectrum of the mixture compared with that of compound 1 indicated the absence of CT interaction in the mixture, assigned to the absence of tpt-triphenylene A–D alignment. The PL decay of the mixture measured at 396 nm and 77 K was fitted by a bi-exponential function with two nanosecond-scale lifetimes (17.49 ns and 50.71 ns, Fig. S33). Meanwhile, the intensities of the decay curves measured at 396 nm decreased as the temperature increased from 77 K to 297 K (Fig. S34). These are typical characteristics of fluorescence. The delayed PL spectra showed that the intensities of the peaks decreased upon increasing the temperature (Fig. S35), and the lifetimes were determined to be 323.01 ms and 2.1 s at 550 nm and 77 K (Fig. S36). According to these results, the peaks at longer wavelengths were assigned to the phosphorescence. These results indicate that the host–guest structure of compound 1 plays an important role in generating intermolecular TSCT interactions and the corresponding TADF.

### X-ray scintillator and imaging applications

X-ray scintillators, which can convert X-rays into low-energy ultraviolet-visible photons, have become a remarkable class of materials widely used for radiation detection, X-ray imaging, medical therapy, *etc.* Specifically, the atoms in the materials interact with X-ray photons through the photoelectric effect and Compton scattering, generating high-energy electrons. These high-energy electrons further interact with atoms in the emitters by thermalizing rapidly, resulting in an avalanche of a tremendous number of secondary electrons and the production of singlet and triplet excitons with a 1 : 3 ratio^12,21,47,48^ (Scheme 1). Finally, the fluorescence/TADF/phosphorescence can be produced by the radiation decay process. Considering the effective utilization of triplet excitons in compound 1 through the RISC process from T_*n*_ to S_*n*_, 1 can be a good candidate as an X-ray scintillator. Moreover, the large Stokes shifts between the absorption and emission spectra originating from the TSCT interaction in compound 1 can lead to weak self-absorption (Fig. 2b and c). These desirable characteristics motivated us to explore the performance of compound 1 as an X-ray scintillator.

**Fig. 4:**
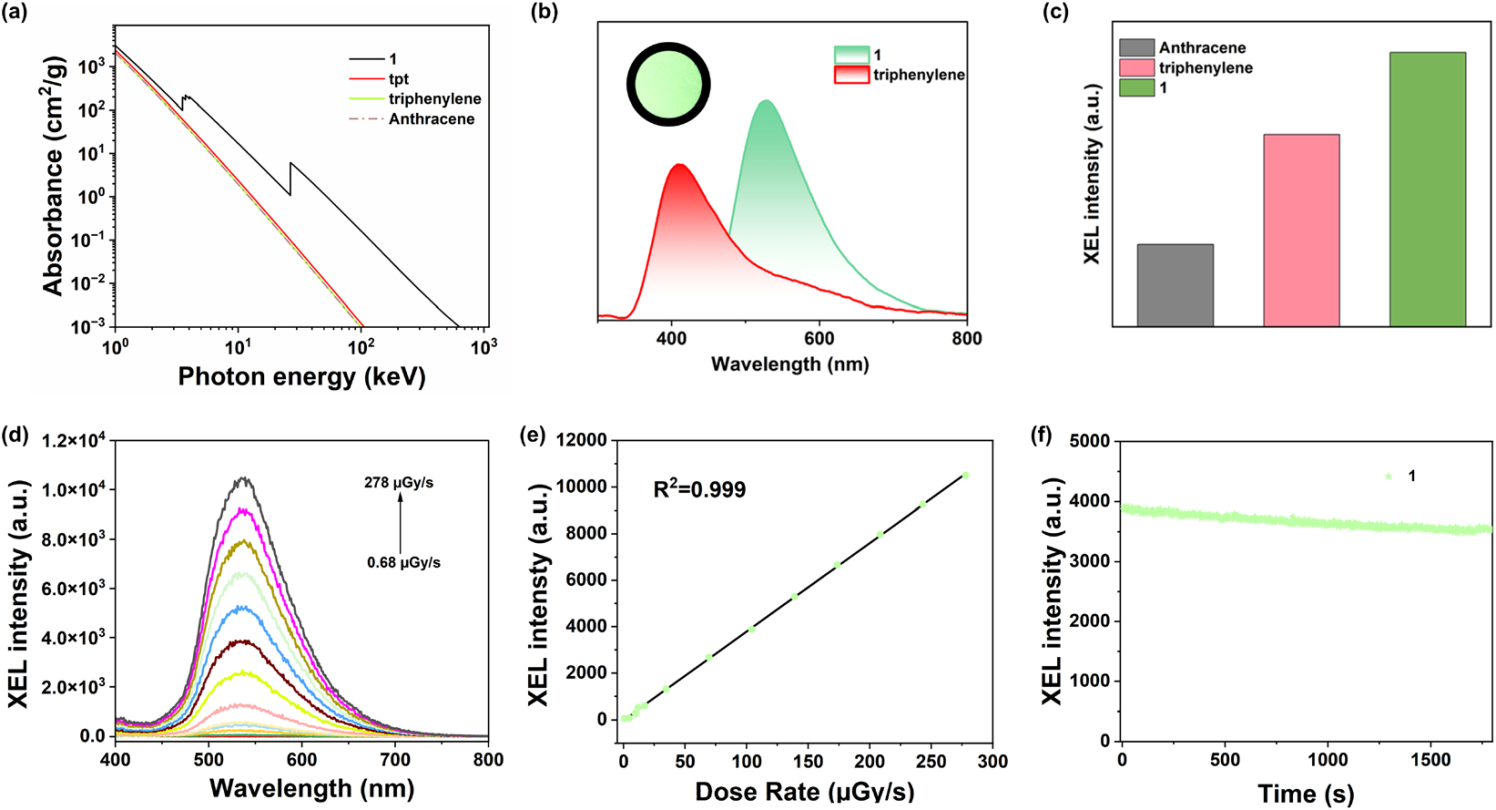
(a) X-ray absorption curves of compound 1, tpt, triphenylene and anthracene; (b) the RL spectra of triphenylene and compound 1 measured by the integrating sphere method, with the inset showing a photograph of compound 1 under X-ray irradiation; (c) the relative RL intensities of anthracene, triphenylene and compound 1; (d) RL spectra of compound 1 at different dose rates; (e) the dose rate dependence of the RL intensity of compound 1; (f) the X-ray irradiation stability of compound 1 at a dose rate of 278 μGy_air_ s^−1^

The simulated X-ray absorption curves were demonstrated to evaluate the X-ray absorption ability of host–guest compound 1. As illustrated in Fig. 4a, compound 1 revealed obviously increased X-ray absorption compared to separate triphenylene and tpt components as well as commercial anthracene, which can be assigned to the TSCT interaction and the heavy atom effect of Cd(ii) centers. Subsequently, the RL spectrum of compound 1 was obtained, which was aligned with the PL spectrum of 1, suggesting the same emission origin. The relative RL intensities of commercial scintillator anthracene, triphenylene, tpt and compound 1 were calculated by integrating the X-ray-induced spectra measured by the integrating sphere method (Fig. 4b and c). The relative RL intensity of triphenylene was 18 964 a.u. while that of tpt cannot be obtained. Upon formation of host–guest MOF 1, an obvious enhancement in RL intensity was observed, reaching up to 23 989 a.u. Besides, the scintillator performance of compound 1 surpassed that of traditional organic commercial scintillator anthracene (6710 a.u.). These results reveal that host–guest TSCT-based efficient TADF emission could enhance exciton utilization and RL performance. The RL spectra of compound 1 were further recorded at X-ray dose rates ranging from 0.68 to 278 μGy_air_ s^−1^ (Fig. 4d), which demonstrated a remarkable linear response to the X-ray dose rates. A linear fitting curve with *R*^2^ = 0.999 was obtained. Using the 3*σ* method, the detection limit of compound 1 was calculated to be 1570 nGy_air_ s^−1^, surpassing the medical diagnostic standard of 5.5 μGy_air_ s^−1^ (Fig. 4e). After continuous exposure to a high X-ray dose (278 μGy_air_ s^−1^) for 30 minutes, the RL intensity remained 90.3%, suggesting a good X-ray photostability (Fig. 4f). All these advantages of compound 1 make it have unique potential for X-ray detection applications.

**Fig. 5:**
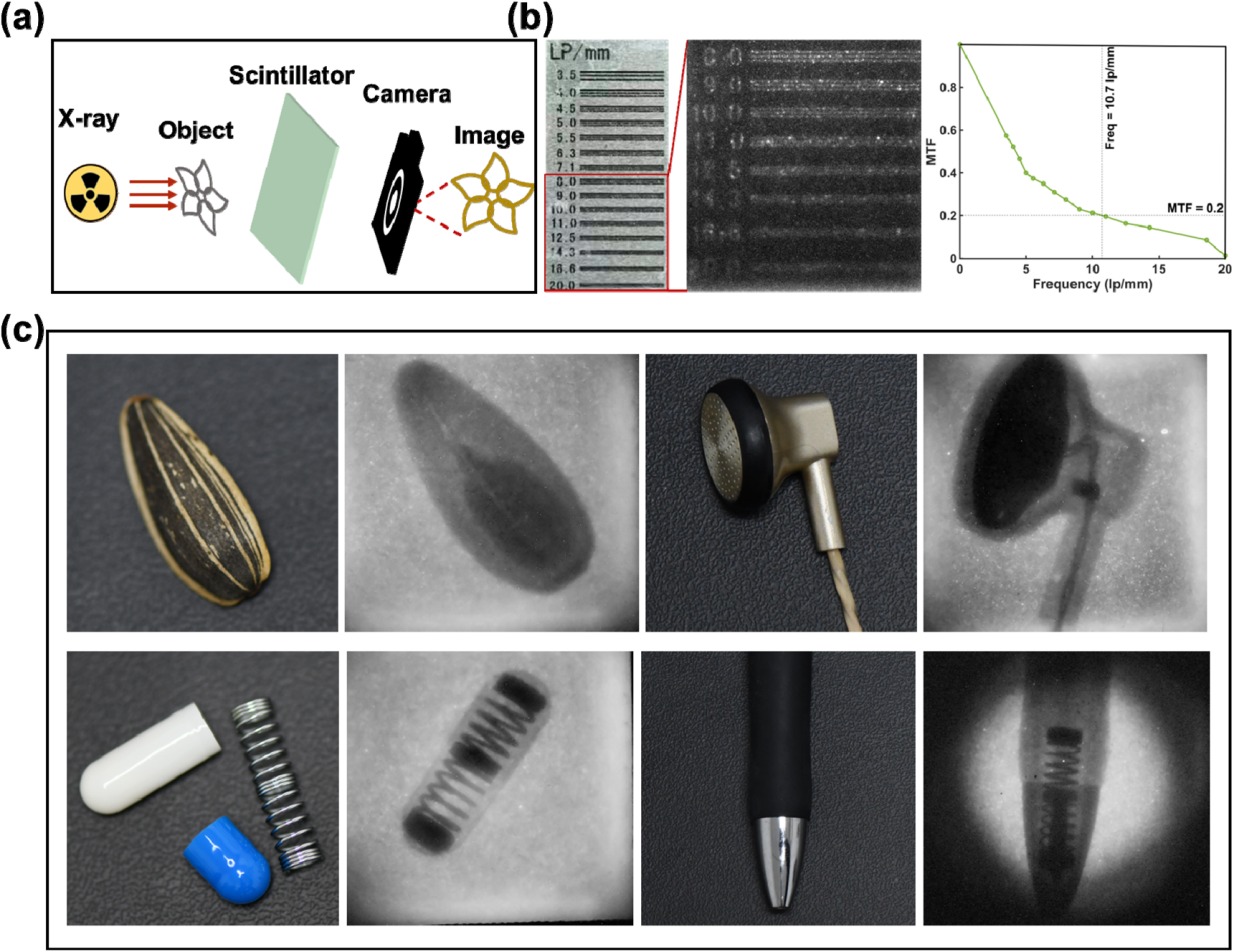
(a) Schematic diagram of the X-ray imaging device; (b) imaging photograph and corresponding resolution of a line pair card and MTF curve; (c) X-ray imaging of a sunflower seed containing lighter elements, a headphone containing the electronics, a capsule and a ballpoint pen containing built-in springs.

Inspired by the promising RL properties, compound 1 was doped into polydimethylsiloxane (PDMS) to prepare a scintillator screen for use in X-ray imaging (for details, see the SI). As shown in Fig. 5a, the test samples were placed between the X-ray source and the scintillator screen and images were captured with a commercial digital camera. Based on the X-ray imaging photo of a standard line pair card, modulation transfer function (MTF) showed the spatial resolution of the scintillator screen to be approximately 10.7 lp mm^−1^ (Fig. 5b). Additionally, the scintillator screen was successfully used for high-quality imaging for real objects such as a sunflower seed containing lighter elements, a headphone containing the electronics, a capsule and a ballpoint pen containing built-in springs (Fig. 5c). These results demonstrate compound 1 as the first example of a TSCT-based TADF host–guest MOF scintillator used for X-ray imaging applications.

## Conclusions

In summary, we proposed a concept of TADF host–guest MOFs with modulational TSCT interactions for X-ray scintillators in this work. By confining D molecules as guests in the pore spaces of host MOFs composed of A ligands, the host–guest MOF materials could be generated. These MOFs exhibited distinct structures owing to the need to match the D–A interactions by an adaptive structural change. Resulting from different TSCT interactions induced by D species, tunable emission wavelengths spanning from 532 nm to 738 nm were achieved. Meanwhile, precise regulation of TSCT states between tpt and triphenylene in compound 1 resulted in TSCT-based TADF properties. The X-ray scintillating properties of compound 1 were further studied. Compound 1 exhibited enhanced scintillator performance compared with that of the separate D and A components as well as that of traditional organic commercial scintillator anthracene. Besides, compound 1 exhibited a detection limit of 1570 nGy_air_ s^−1^. To the best of our knowledge, this is the first example of a TSCT-based TADF host–guest MOF scintillator used for X-ray imaging applications. These findings provide a general strategy for developing efficient host–guest MOF scintillators towards high-performance X-ray detection and imaging.

## Author contributions

X. Yan performed experiments, analysed the data and wrote the supporting information. S.-Y. Song recorded the RL spectra supervised by K.-K. Liu. S. Liang synthesized parts of samples. X.-T. Liu wrote the original draft of the manuscript. X.-T. Liu and C. Lu conceptualized and supervised this work. All authors contributed to reviewing and editing the manuscript.

## Conflicts of interest

There are no conflicts to declare.

## Supplementary Material

SC-OLF-D5SC02235E-s001

SC-OLF-D5SC02235E-s002

## Data Availability

CCDC 2413460 (1), 2413463 (2) and 2413462 (3) contain the supplementary crystallographic data for this paper.^49–51^ The data supporting the findings of this study are available within the article and SI. See DOI: https://doi.org/10.1039/d5sc02235e. All other relevant source data are available from the corresponding authors upon reasonable request.
