# Supplementary material for: Regulating through-space charge transfer interactions in donor–acceptor MOFs for thermally activated delayed fluorescence and X-ray scintillators
Source: Chem Sci. 2025 Aug 12;16(36):16770–9. doi: 10.1039/d5sc02235e (PMC12365922; doi:10.1039/d5sc02235e)
Supplement: SC-016-D5SC02235E-s001 [file SC-016-D5SC02235E-s001.pdf]

Supplementary Information (SI) for Chemical Science.
This journal is © The Royal Society of Chemistry 2025

Regulating Through-Space Charge Transfer Interactions in Donor-Acceptor MOFs for Thermally Activated Delayed Fluorescence and X-ray Scintillator

Xinyue Yan[a], Shi-Yu Song[b], Shicong Liang[a], Kai-Kai Liu[b], Xiao-Ting Liu*[a], and Chao Lu*[a,c]

[a]*College of Chemistry, Pingyuan Laboratory, Zhengzhou University, Zhengzhou, 450001, China.*

[b]*Henan Key Laboratory of Diamond Optoelectronic Material and Devices, Key Laboratory of Material Physics, Ministry of Education, School of Physics and Microelectronics, Zhengzhou University, Zhengzhou 450001, China*

[c]*State Key Laboratory of Chemical Resource Engineering, Beijing University of Chemical Technology, Beijing, 100029, China.*

*Corresponding author:

liuxiaoting@zzu.edu.cn (X.-T. Liu), luchao@mail.buct.edu.cn (C. Lu).

Postal address: College of Chemistry, Pingyuan Laboratory, Zhengzhou University, Zhengzhou, 450001, China.

Section 1. Experimental Sections

1. Materials and Instruments

The raw materials and reagents used were commercially purchased, and no further purification was required.

Single crystal X-ray diffraction (SCXRD) experiment of compound **1** was recorded on a Rigaku XtaLAB AFC12 (RINC): Kappa single diffractometer with Cu Kα radiation (λ=1.54184 Å) at 120 K. SCXRD experiment of compound **2** was recorded on a Rigaku XtaLAB Synergy R, HyPix diffractometer with Cu-Ka radiation (λ=1.54184 Å) at 100 K. SCXRD of compound **3** was recorded on a Rigaku XtaLAB AFC12 (RINC): Kappa single diffractometer with Cu-Ka radiation (λ=1.54184 Å) at 299 K. The structures were solved by intrinsic phasing methods using SHELXL program. Refining programs were performed using SHELXL-2019/2, and final full-matrix refinements were against F^2 [1]. The crystallographic data and structure refinements for compounds **1**-**3** were listed in Table S1 and the selected bond lengths and angels of compounds **1**-**3** were listed in Tables S9-S11. The CCDC numbers are 2413460, 2413463 and 2413462, respectively.

The powder X-ray diffraction (PXRD) measurements were performed with a Rigaku MiniFlex600 diffractometer with Cu-Kα radiation (λ=1.54184 Å). Variable-temperature PXRD were performed on a Rigaku SmartLab diffractometer, equipped with a TTK 600 low-temperature chamber to control the temperature. A turbo vacuum pump can be further connected with the chamber to provide a vacuum atmosphere. Thermogravimetric analysis (TGA) was performed on a Setline STA thermal analyzer. Nuclear magnetic resonance (^{1}H NMR) data were obtained on a Bruker Avance III HD 400 M spectrometer by digesting these crystalline samples in a mixed solution (trifluoroacetic acid-d and dimethyl sulfoxide-d_6). Elemental analysis was recorded by elementar UNICUBE. The UV-vis spectra were recorded by Shimadzu UV-2700i spectrophotometer. The PL spectra of the solid samples at room temperature and temperature-dependent PL spectra were measured with an Edinburgh FLS1000 fluorescence spectrophotometer. Temperature-dependent transient PL decay curves in the millisecond and microsecond regions were measured with the FLS1000

spectrophotometer and a microsecond flash-lamp as an excitation source. Temperature-dependent transient PL decay curves in the nanosecond region were obtained by the Edinburgh FLS1000 instrument with a 365 nm laser as the excitation source. The absolute photoluminescence quantum yields (PLQYs) were determined by the Edinburgh FLS1000 spectrophotometer using an integrating sphere. Radioluminescence (RL) spectra were recorded by an Edinburgh FS5 fluorescence spectrophotometer equipped with an external miniature X-ray source. X-ray imaging was performed by a homemade setup with the use of an external miniature X-ray tube, a crystalline sample doped film, and a digital camera as excitation, scintillation screen, and detector, respectively.

2. Synthesis of 1-3

For the synthesis of **1**, $Cd(NO_3)_2 \cdot 4H_2O$ (15.4 mg, 0.05 mmol), tpt (15.6 mg, 0.05 mmol), H_2bpdc (12.1 mg, 0.05 mmol), triphenylene (11.4 mg, 0.05 mmol), and 4 mL DMF, 2 mL EtOH, 2 mL H_2O and 150 μL HBF_4 were mixed in a 20 mL glass bottle, sealed, and sonicated to the bottom without obvious precipitation. The bottle was heated at 92 °C for 24 h to obtain yellow-green crystal **1**. Elemental analysis calculated for $C_{50}H_{34}CdN_6O_5$: C 65.93%, H 3.74%, N 9.23%; Found C 65.61%, H 3.75%, N 9.26%.

For the synthesis of **2**, $Cd(NO_3)_2 \cdot 4H_2O$ (15.4 mg, 0.05 mmol), tpt (15.6 mg, 0.05 mmol), H_2bpdc (12.1 mg, 0.05 mmol), perylene (10 mg), and 4 mL DMF, 2 mL EtOH, 2 mL H_2O were mixed in a 20 mL glass bottle, sealed, and sonicated to the bottom without obvious precipitation. The bottle was heated at 92 °C for 48 h to obtain deep-red crystal **2**. Elemental analysis calculated for $C_{104}H_{68}Cd_2N_{12}O_{10}$: C 66.78%, H 3.64%, N 8.99%; Found C 66.56 %, H 3.55 %, N 8.96 %.

For the synthesis of **3**, $Cd(NO_3)_2 \cdot 4H_2O$ (15.4 mg, 0.05 mmol), tpt (15.6 mg, 0.05 mmol), H_2bpdc (12.1 mg, 0.05 mmol), and coronene (3.3 mg), and 4 mL DMF, 2 mL EtOH, 2 mL H_2O and 150 μL HBF_4 were mixed in a 20 mL glass bottle, sealed, and sonicated to the bottom without obvious precipitation. The bottle was heated at 92 °C for 48 h to obtain yellow crystal **3**. Elemental analysis calculated for $C_{56}H_{34}CdN_6O_5$: C 68.40%, H 3.46%, N 8.55%; Found C 68.12%, H 3.45%, N 8.62%.

3. X-ray dosage detection

The radiation dose of X-ray is controlled by changing the current and voltage of miniature X-ray source. The RL spectra of **1** were obtained with varied X-ray dosage from 0.68 $\mu Gy_{air}\ s^{-1}$ to 278.0 $\mu Gy_{air}\ s^{-1}$. The relationship between the current and voltage of miniature X-ray source and the corresponding radiation dose was shown in Table S8. The limit of detection (LOD) was calculated by LOD = $3\sigma/ k$, where σ is the standard deviation calculated by repeated tests of background signals for ten times, and k is the slope of linear fitting curve.

4. Preparation of scintillation screen

The sample of **1** (5 wt %) was uniformly dispersed in the *n*-hexane solution, and then added to the premixed polymer matrix (SYLGARD 184 silicone elastomer:curing agent=2:1, mass ratio). The mixture was stirred vigorously to form the mixture gel. The gel mixture was placed in a Plexiglas mold (3 cm×3 cm×1 mm) and heated for 2 h at 80 °C using a hot platform. When the mixture gel was cooled to room temperature, it was peeled from the mold to form smooth and flat scintillator films.

5. **X-ray luminescence imaging**

We have constructed a high-resolution X-ray imaging system consisting of a miniature x-ray source, a scintillator film, and a digital camera. The test samples were placed between the X-ray source and the scintillator film, and photos were collected from the other side of the scintillator film using a digital camera. The operating voltage and current of the micro-X-ray light tube are 0-60 kV and 0-200 µA, respectively.

Section 2. Computational Details

Theoretical calculations were carried out by using the Quickstep algorithm of *CP2K (version 2023.2)* package[2]. Three-dimensional simplified models of **1, 2** and **3** were extracted from crystal structures with periodically stacked guests and ligands. The simplified models maintain the necessary supramolecular between tpt ligands and guests, and require less computation cost than the whole crystal structures. These periodic structures were first optimized to an energy-minimal geometry (only hydrogen were optimized and other elements were restrained), and computed both singlet and triplet excitation states. Subsequently, the excited states of these structures were optimized to an energy-minimal geometry with the computation of both singlet and

triplet excitation states. The structural optimizations were performed using Perdew-Burke-Ernzerhof (PBE) functional[3,4] with the Grimme's dispersion correction with Becke-Johnson damping (D3BJ)[5,6]. The excited state was calculated using a more expensive hybrid functional PBE0[7] for better accuracy. The Gaussian and augmented plane wave scheme[8], a Pople basis set 6-31G* and a 350 Ry plane wave cutoff for the auxiliary grid were used. The periodic decoupling method was employed to solve the Poisson equation. A convergence criterion of 3.0×10^{-6} a.u. was used for the optimization of the wave function. The input/output file process, generation of electron transition density maps, and analysis of interfragment charge transfer (IFCT) were supported by *Multiwfn (version 3.8dev)* package[9,10].

Section 3. Supplementary Figures and Tables

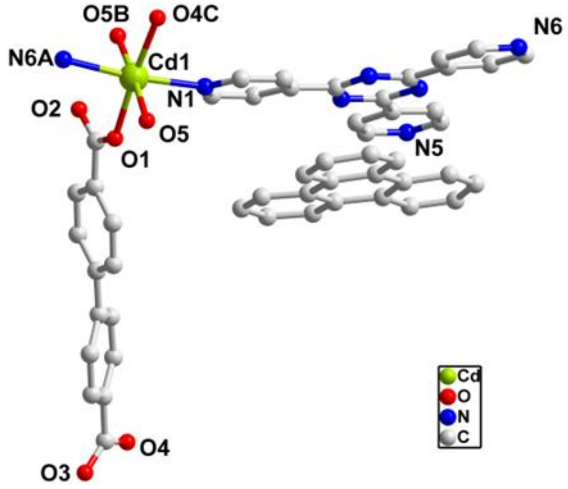

Figure S1. The asymmetric unit of **1**. Color codes: green Cd, red O, gray C, blue N.

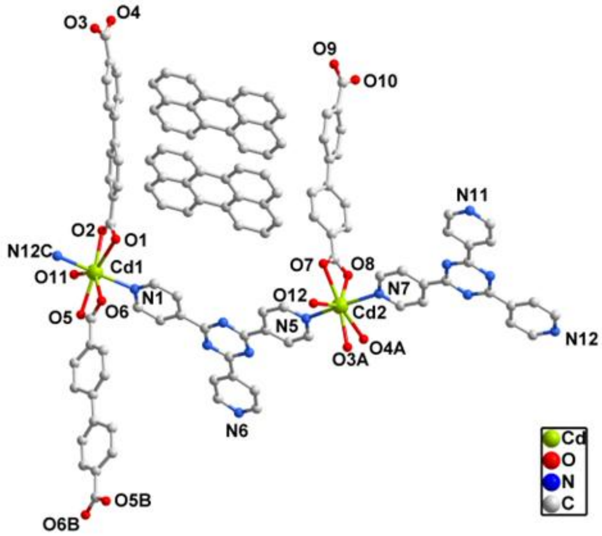

Figure S2. The asymmetric unit of **2**. Color codes: green Cd, red O, gray C, blue N.

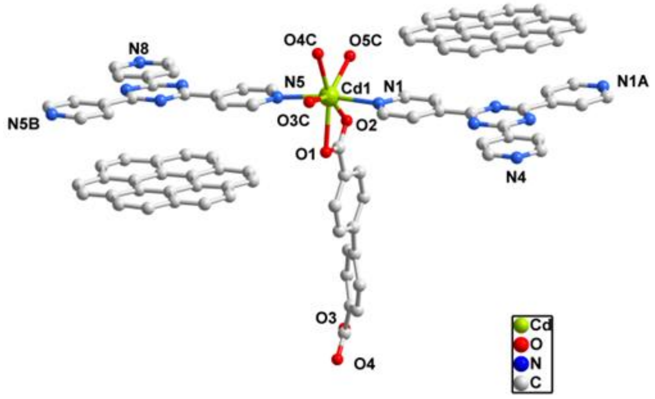

Figure S3. The asymmetric unit of **3**. Color codes: green Cd, red O, gray C, blue N.

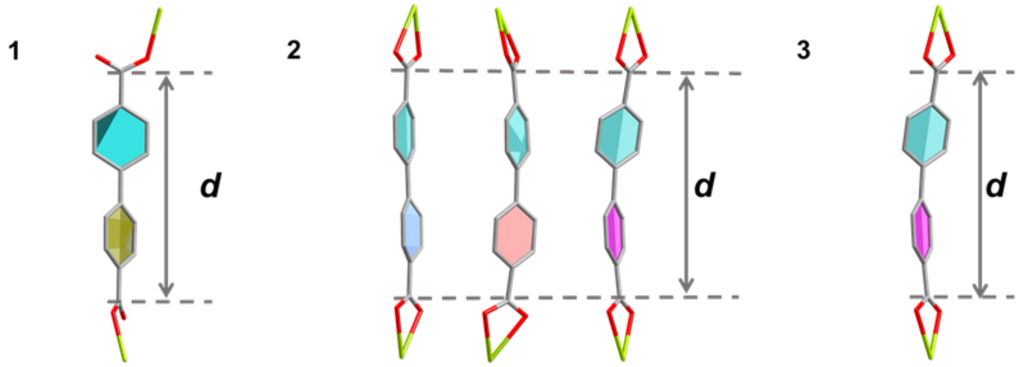

Compound	The coordination pattern of the bpdc^{2-}	$d_{(C\text{-}C)}$	The dihedral angles of the planes of the two benzene rings in the bpdc^{2-}
1	1	10.1252(23)	42.089(52)
2	3	10.1414(1)<br>10.0704(1)<br>10.0452(1)	0.001<br>22.771<br>32.760
3	1	10.0261(123)	38.391(291)

Figure S4. The coordination patterns of the bpdc^{2-} in **1**, **2** and **3** (top); The distances of carboxyl carbon atoms and the dihedral angles of the planes of the two benzene rings in the corresponding bpdc^{2-} ligands (bottom).

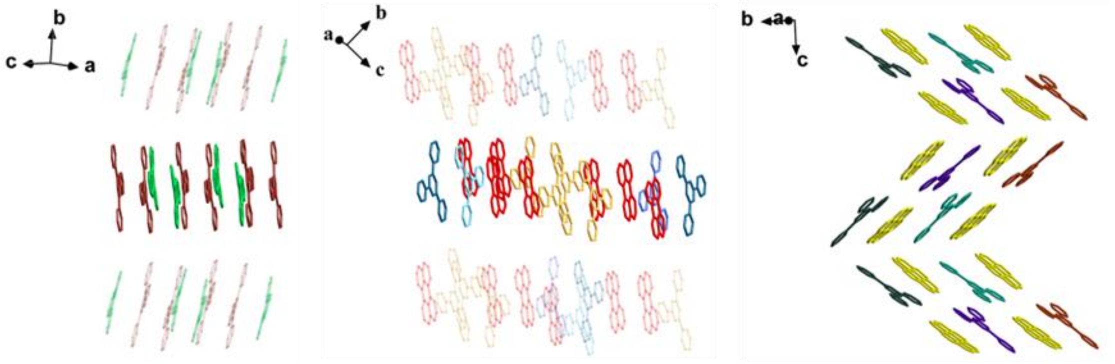

Figure S5. The stacking modes of tpt and corresponding D guests in **1** (left), **2** (middle) and **3** (right).

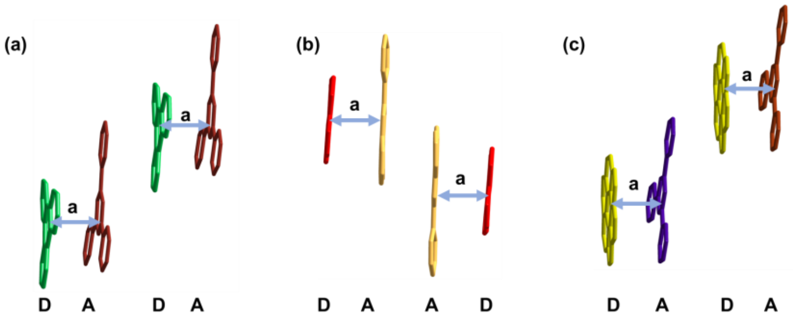

(d)

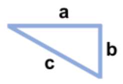

	1	2	3
a (Å)	3.2733	3.3864	3.5048
b (Å)	1.3054	1.4216	1.1373
c (Å)	3.5240	3.6727	3.6847

Figure S6. (a)-(c) The vertical distances between A (tpt ligand) and D (PAH guests) in compounds **1-3**; (d) Simplified model for comparing relative positions between A and D guests (a represents the vertical distances between D and A responsive for the TSCT interactions; b represents the distances between the projection point for center A to the D plane and the center of D, which reflects the areas of face-to-face stacking to some degree; c represents the distances between center A and center D).

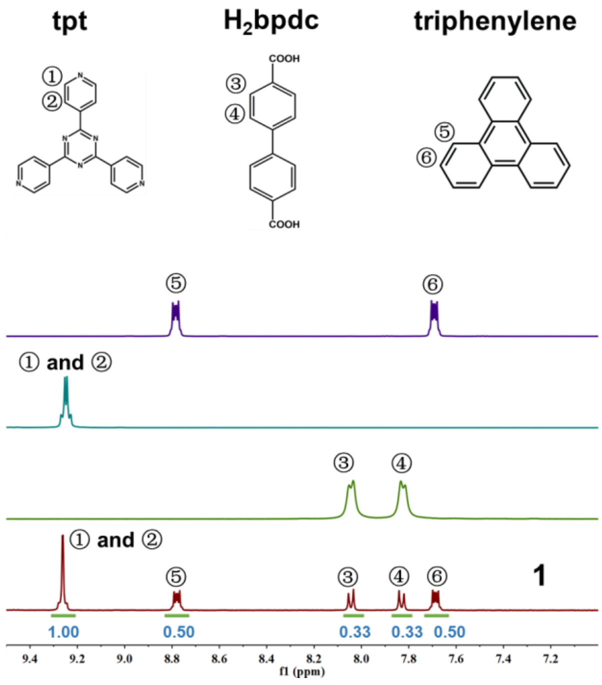

Figure S7. The ^{1}H NMR spectra of digested **1**, the corresponding ligands and triphenylene.

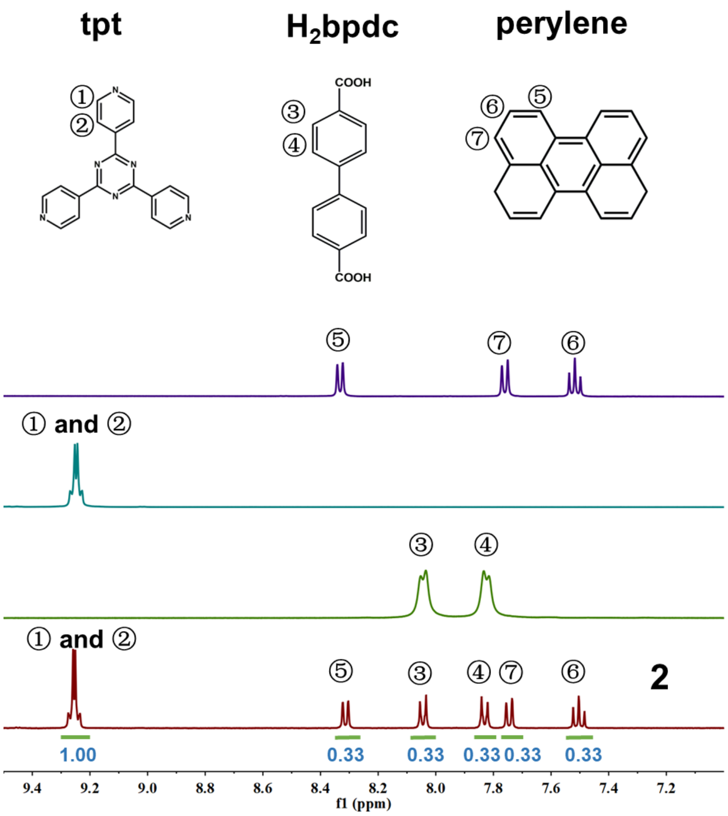

Figure S8. The ^{1}H NMR spectra of digested **2**, the corresponding ligands and perylene.

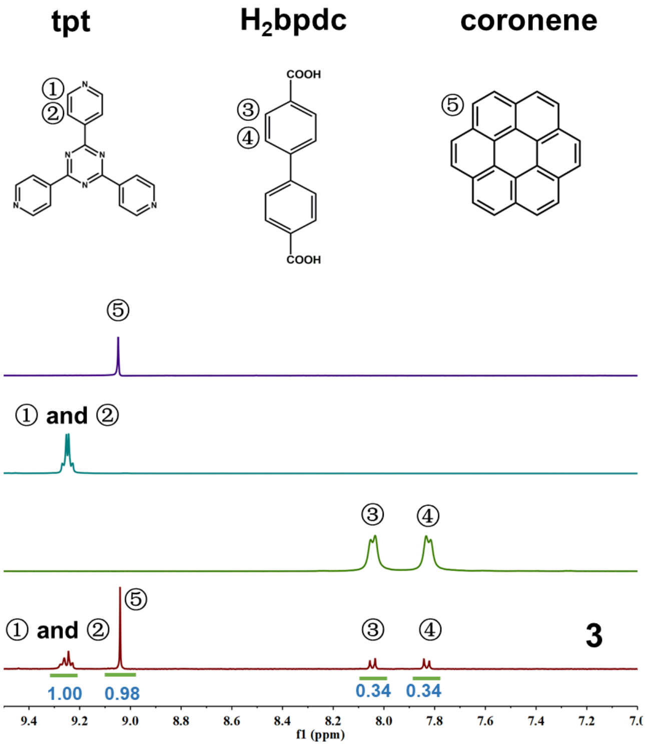

Figure S9. The ^{1}H NMR spectra of digested **3**, the corresponding ligands and coronene.

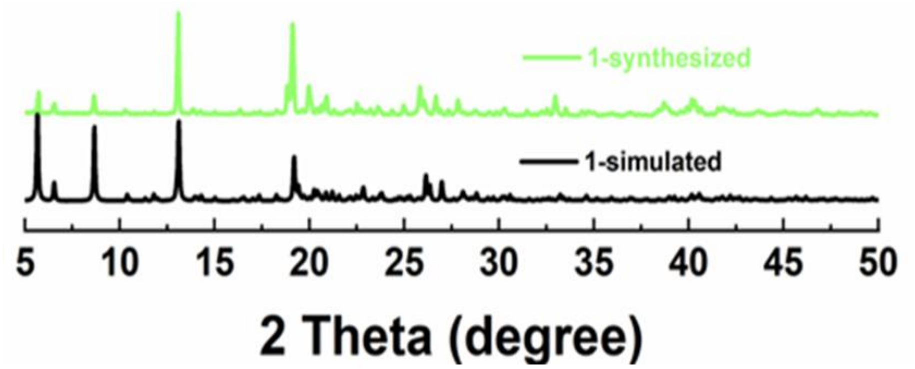

Figure S10. The simulated and as-synthesized PXRD patterns of **1**.

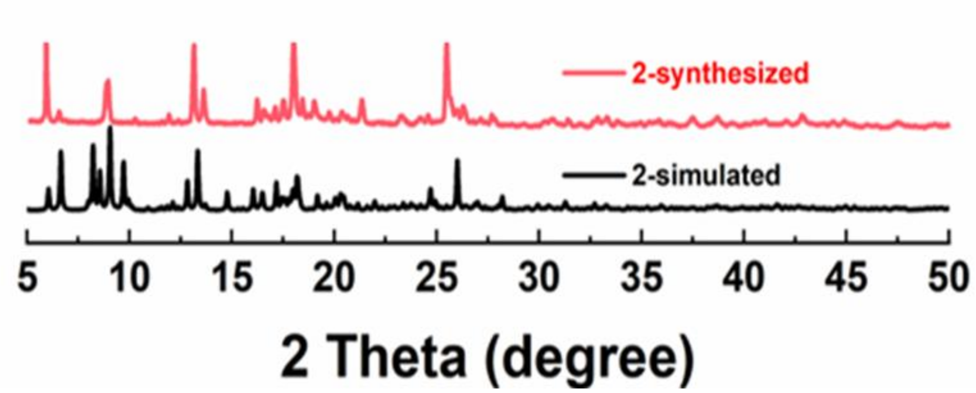

Figure S11. The simulated and as-synthesized PXRD patterns of **2**.

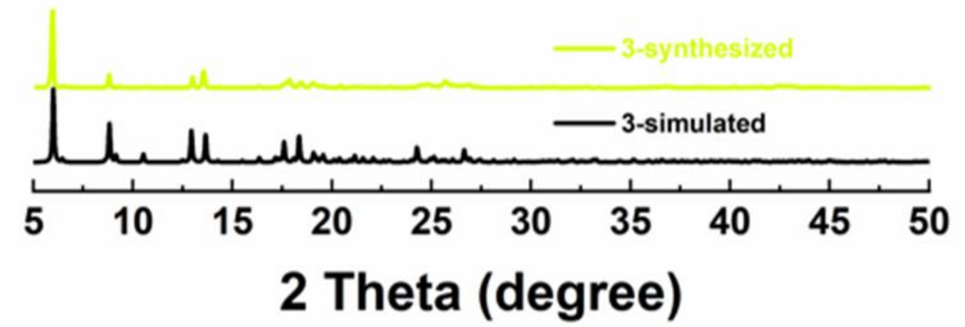

Figure S12. The simulated and as-synthesized PXRD patterns of **3**.

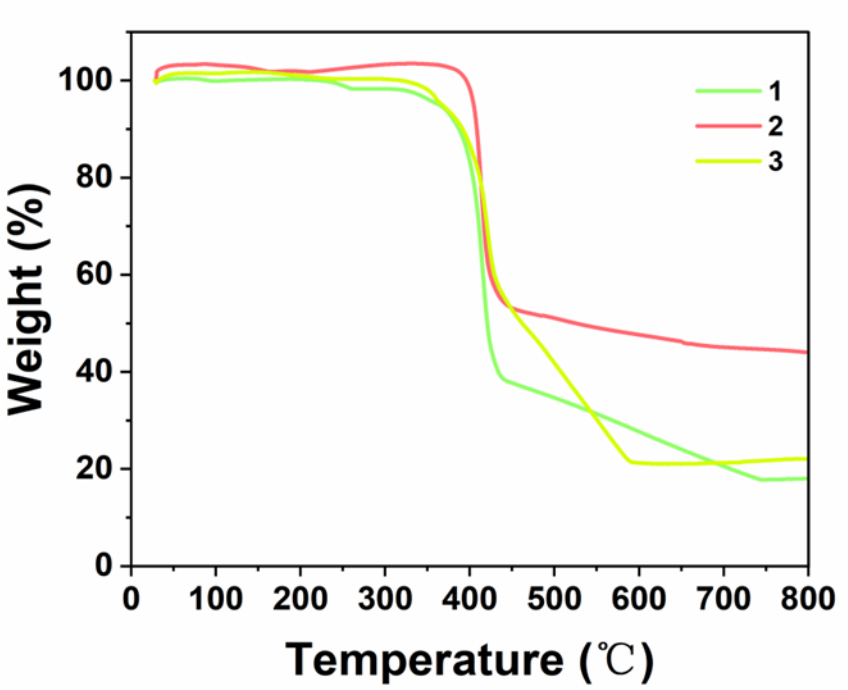

Figure S13. The TG curves of **1-3** at air atmosphere.

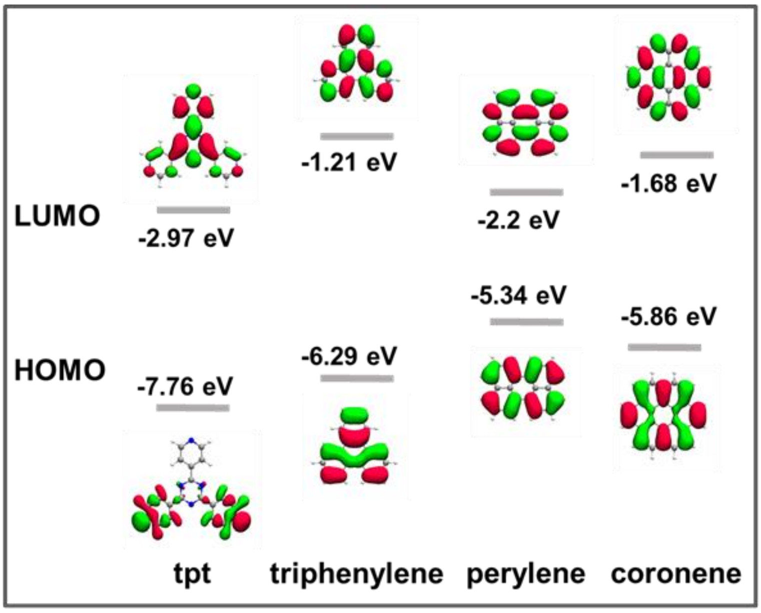

Figure S14. HOMO/LUMO distributions of tpt ligand and guest molecules.

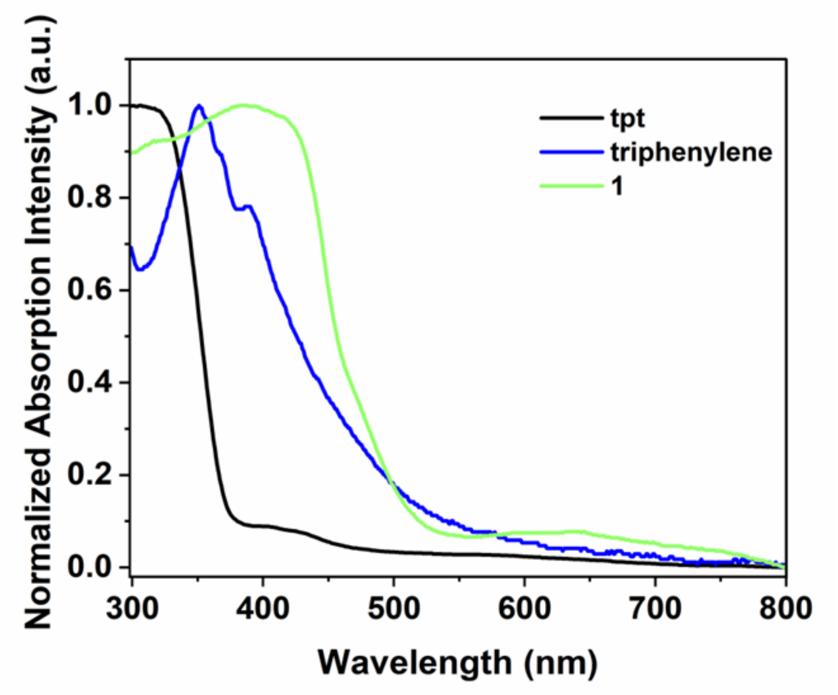

Figure S15. UV-vis absorption spectra of the tpt ligand, triphenylene and **1**.

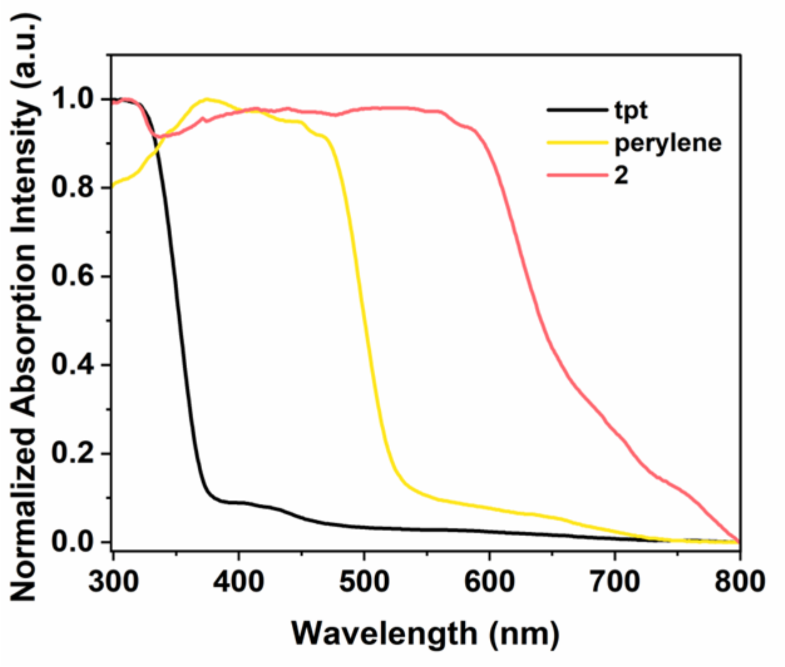

Figure S16. UV-vis absorption spectra of the tpt ligand, perylene and **2**.

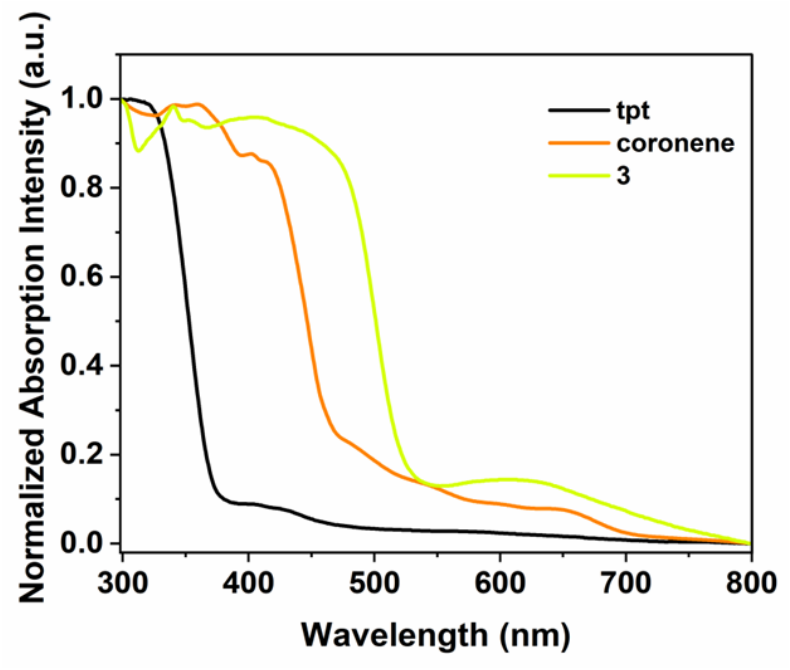

Figure S17. UV-vis absorption spectra of the tpt ligand, coronene and **3**.

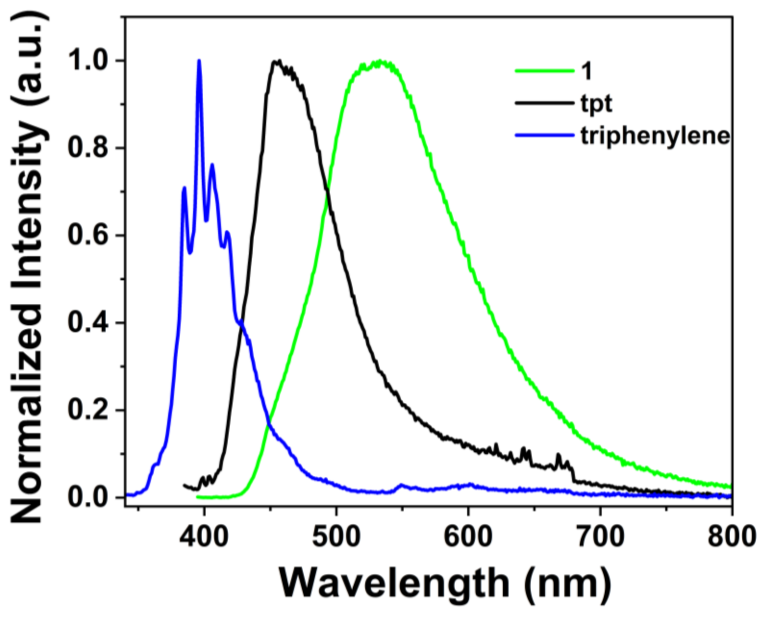

Figure S18. The PL spectra of the tpt ligand, triphenylene and **1**.

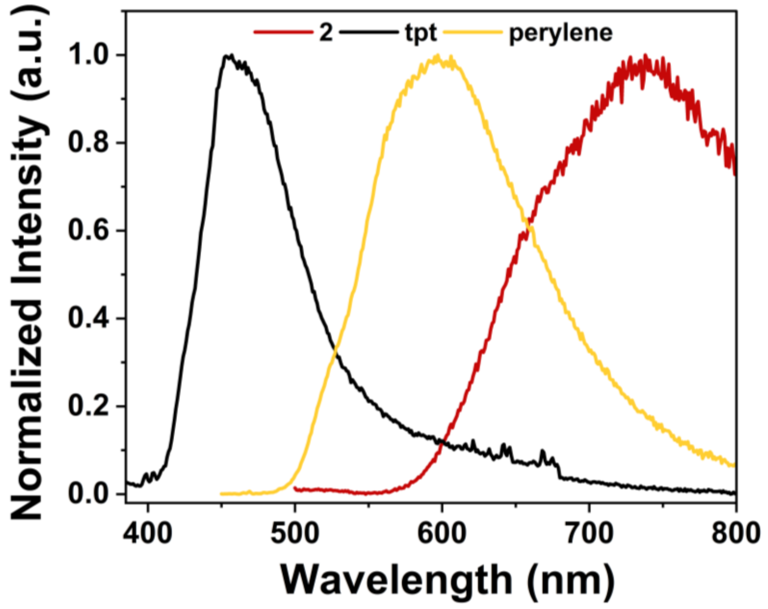

Figure S19. The PL spectra of the tpt ligand, perylene and **2**.

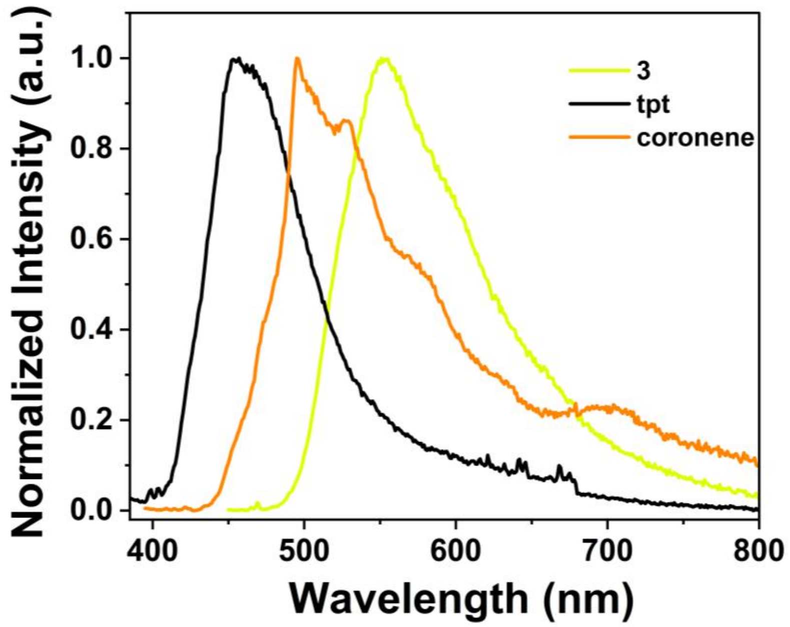

Figure S20. The PL spectra of the tpt ligand, coronene and **3**.

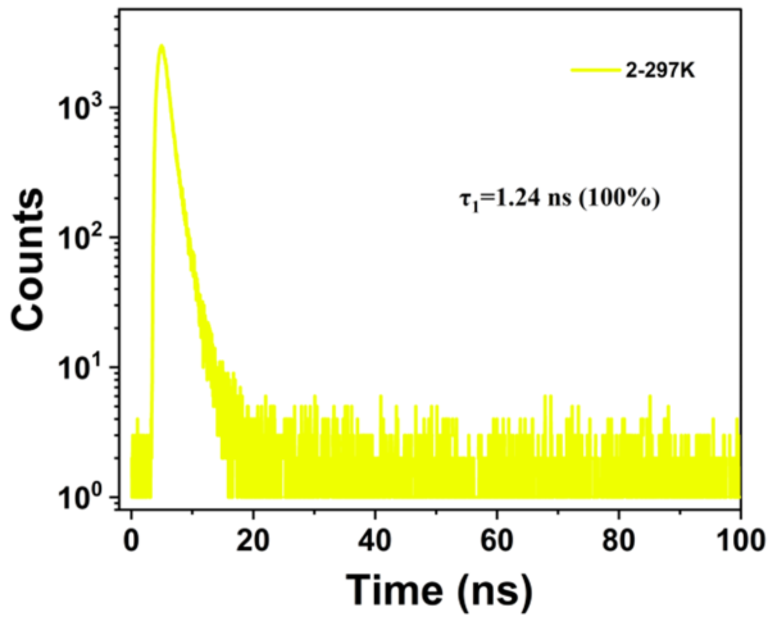

Figure S21. The transient PL decay curve of **2** at 738 nm and 297 K.

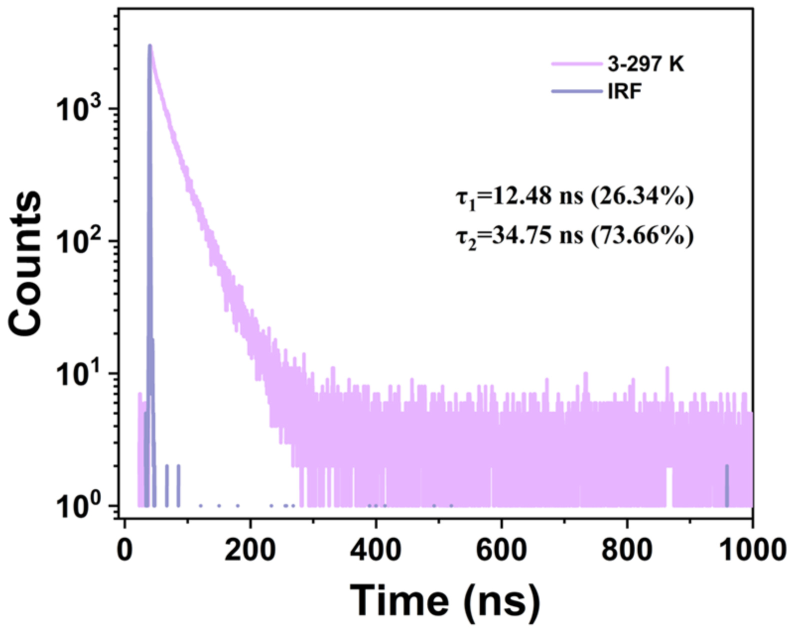

Figure S22. The transient PL decay curve of **3** at 553 nm and 297 K.

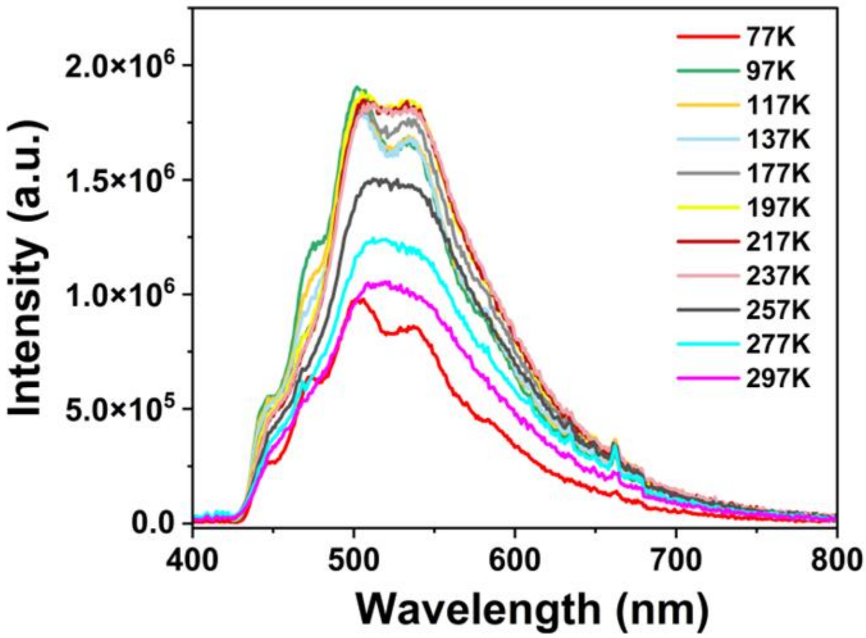

Figure S23. Variable-temperature PL spectra of **1**.

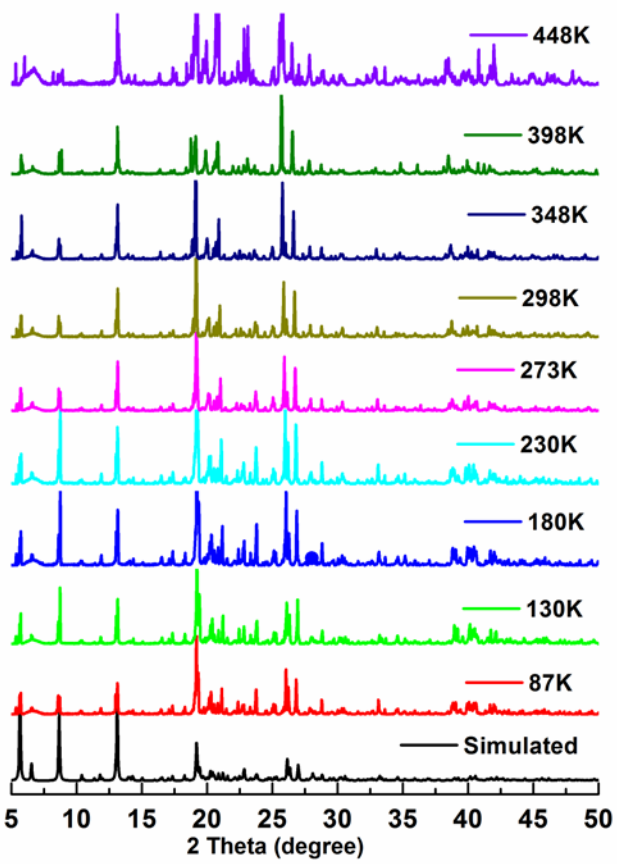

Figure S24. Variable-temperature PXRD patterns of **1**.

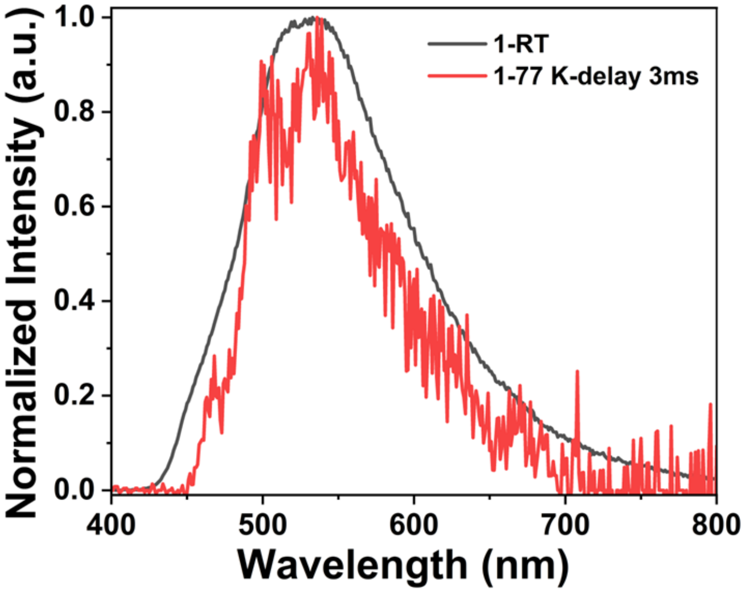

Figure S25. The steady-state PL spectrum at 298 K and delayed PL spectrum at 77 K of compound **1**.

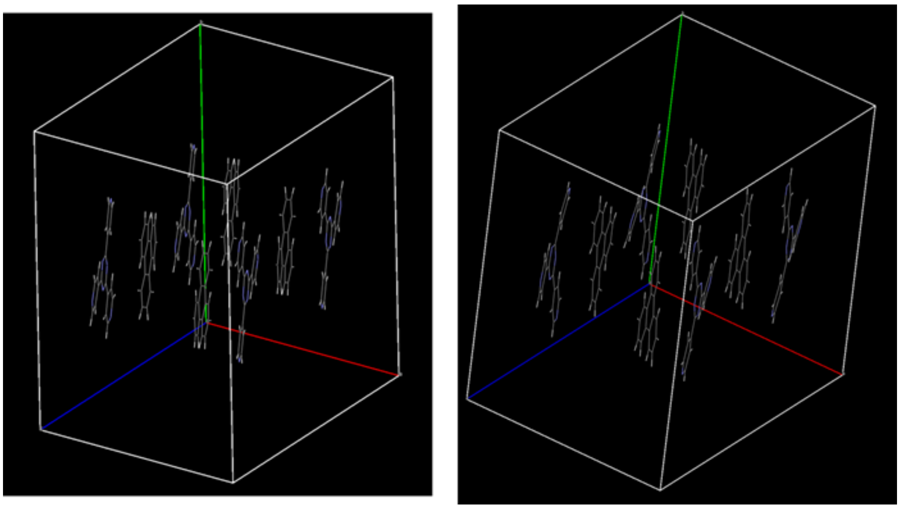

Figure S26. The structural models of ground geometry of compound **1** (left, drawn from crystal structure) employed for DFT calculation and the corresponding relaxed excited state geometry (right).

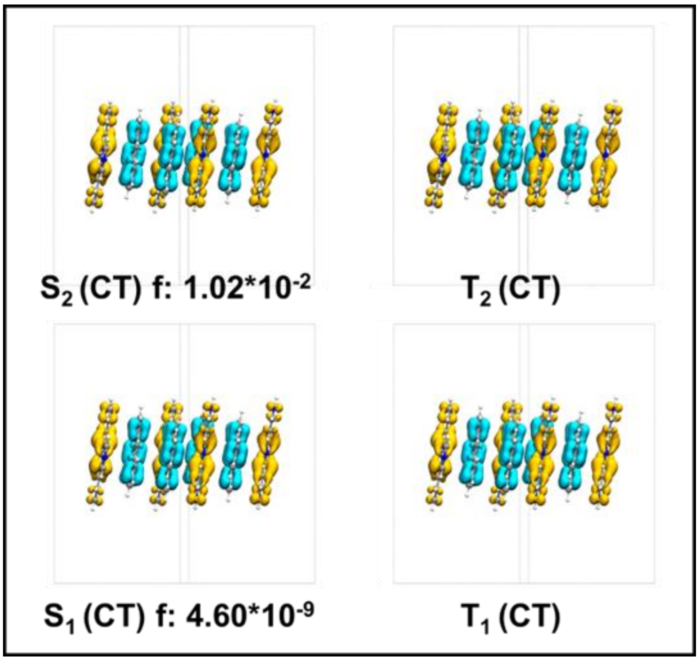

Figure S27. Electron transition density analysis for the two lowest singlet/triplet states at ground geometry for compound **1**, all of which were characterized as CT states (blue, hole; yellow, electron; isovalue: 0.0004).

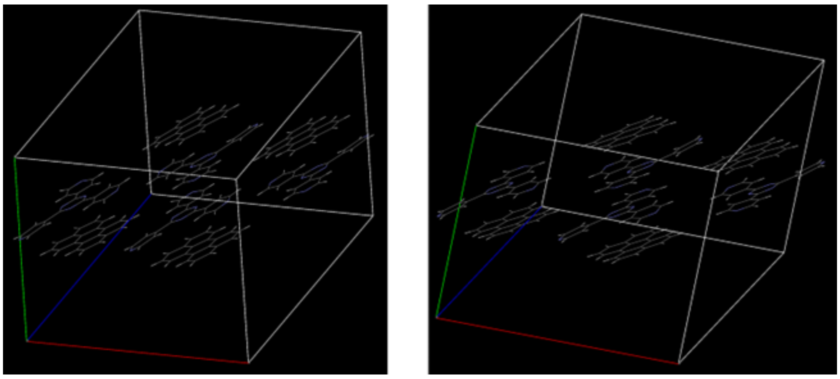

Figure S28. The ground geometry (left) and relaxed excited states geometry (right) of compound **2**.

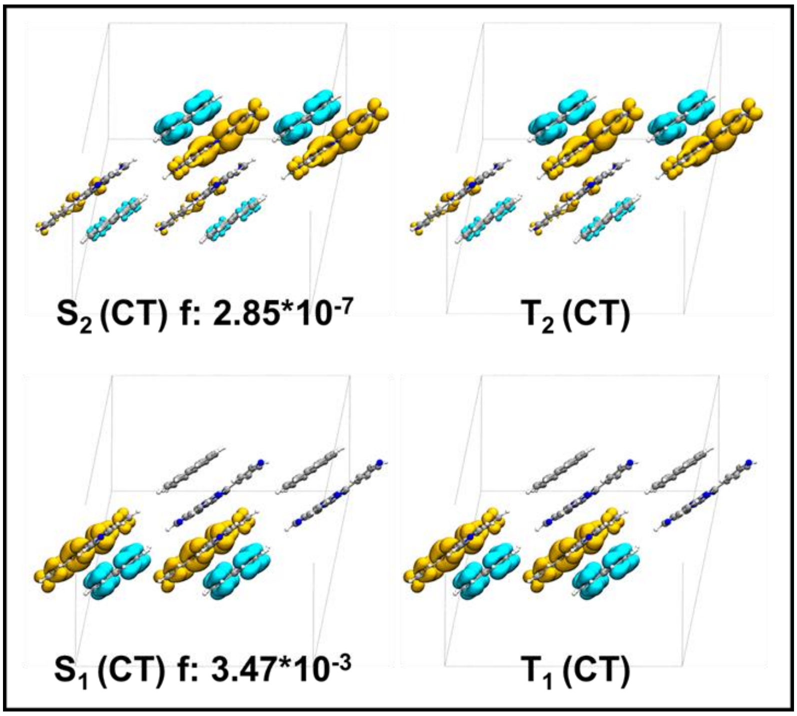

Figure S29. Electron transition density analysis for the two lowest singlet/triplet states at ground geometry for compound **2**, all of which were characterized as CT states (blue, hole; yellow, electron; isovalue: 0.0004).

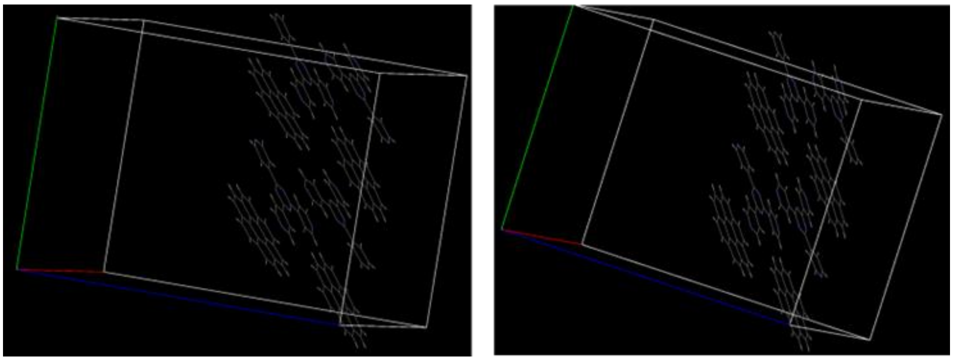

Figure S30. The ground geometry (left) and optimized excited state geometry (right) of compound **3**.

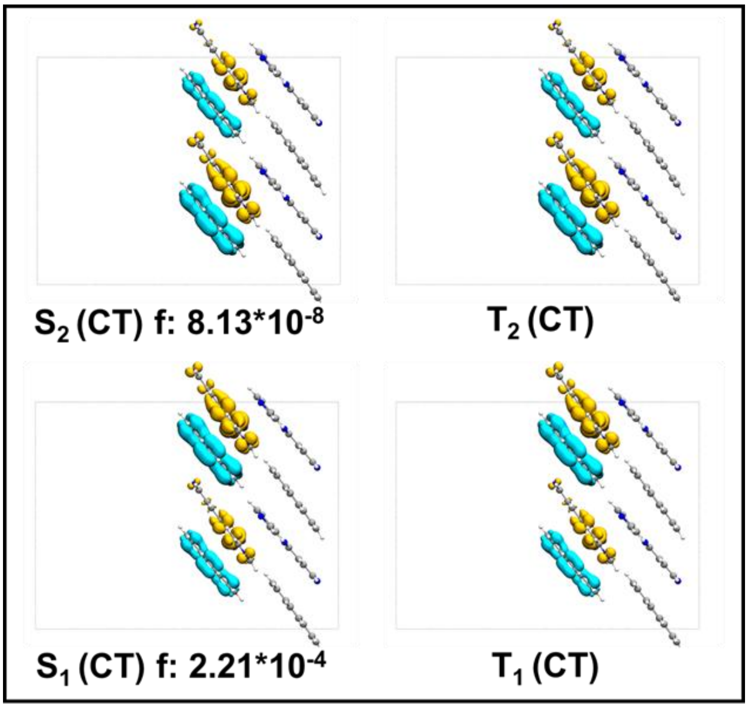

Figure S31. Electron transition density analysis for the two lowest singlet/triplet states at ground geometry for compound **3**, all of which were characterized as CT states (blue, hole; yellow, electron; isovalue: 0.0004).

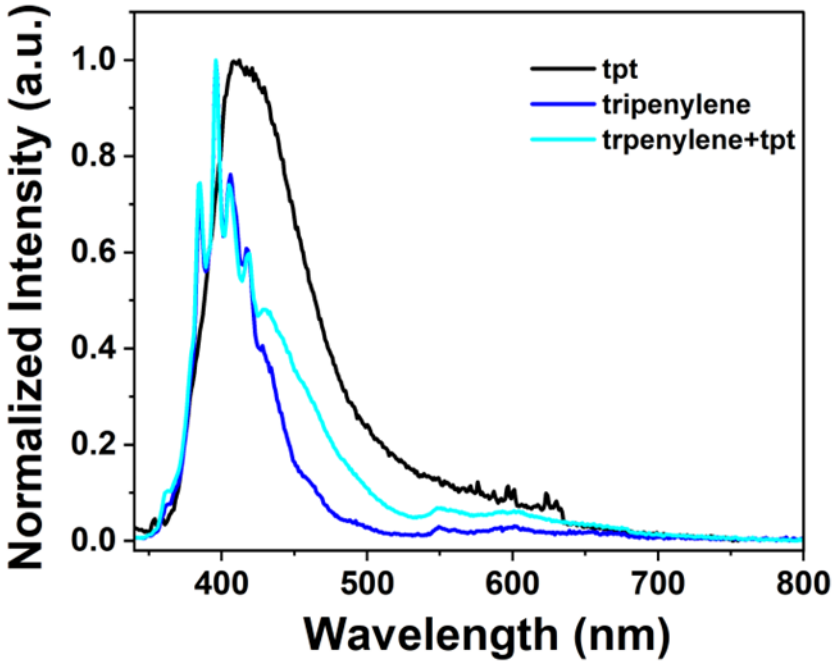

Figure S32. The PL spectra of tpt, triphenylene and the mixture of tpt and triphenylene.

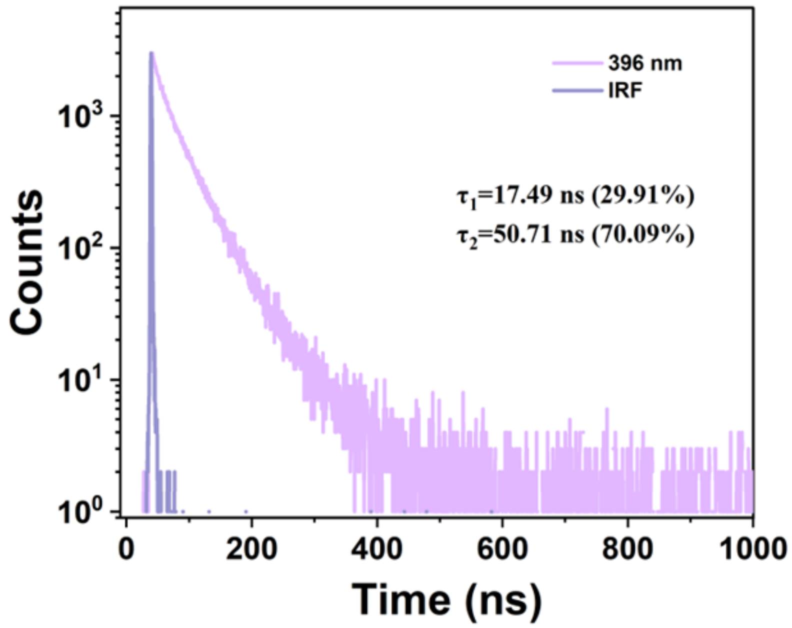

Figure S33. The transient PL decay curve of the mixture of tpt and triphenylene at 396 nm and 77 K.

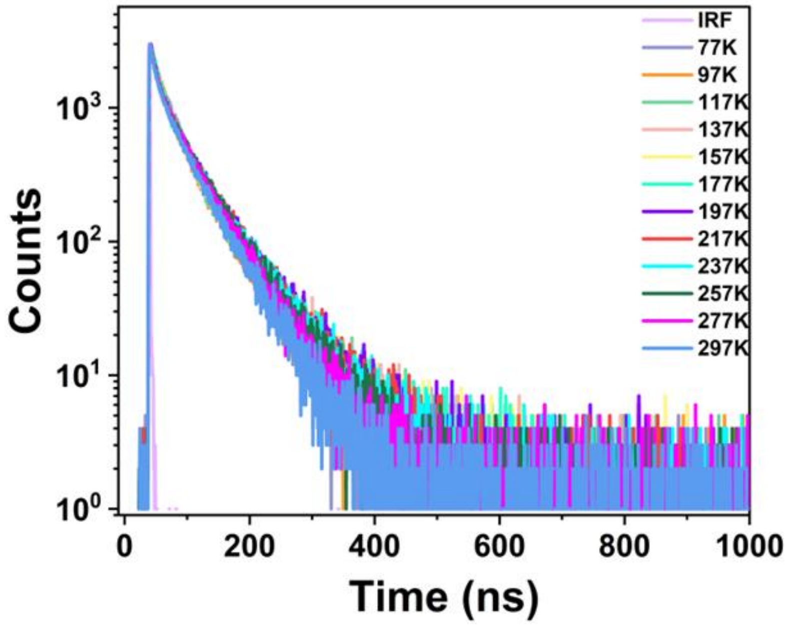

Figure S34. The temperature-dependent PL decay curves of the mixture of tpt and triphenylene at 396 nm.

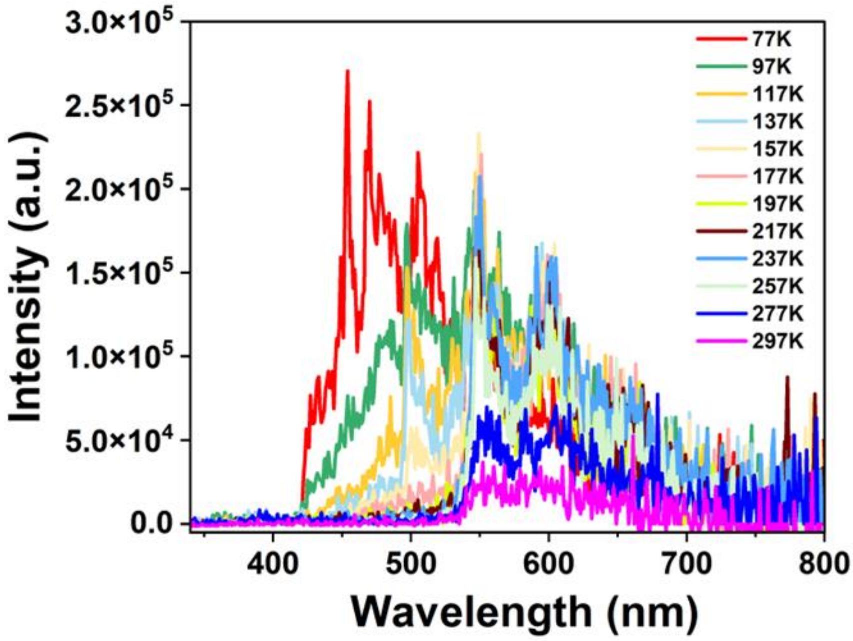

Figure S35. Delayed PL spectra of the mixture of tpt and triphenylene at different temperatures.

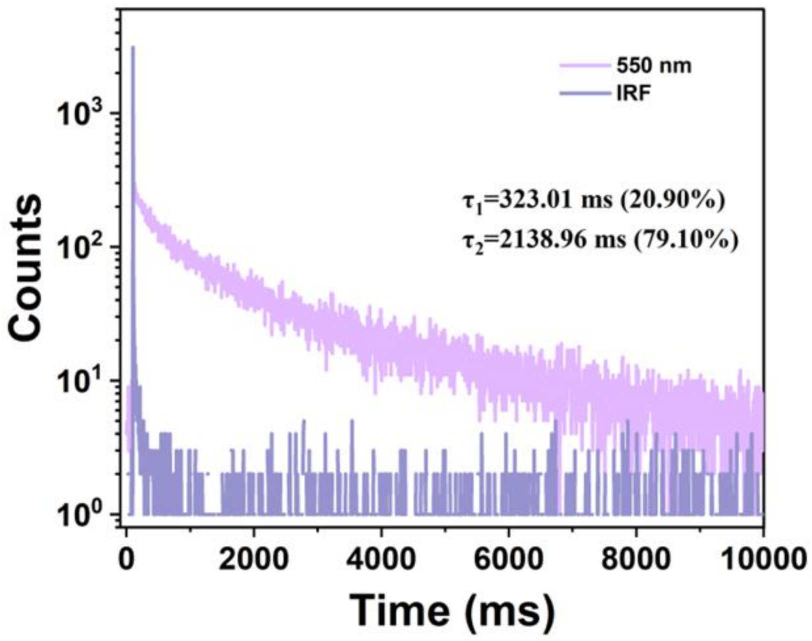

Figure S36. The transient PL decay curve of the mixture of tpt and triphenylene at 550 nm and 77 K.

Table S1 Crystal data and structure refinement for compounds **1-3**.

**Compounds**	**1**	**2**	**3**
Empirical formula	$C_{50}H_{34}CdN_6O_5$	$C_{104}H_{68}Cd_2N_{12}O_{10}$	$C_{56}H_{34}CdN_6O_5$
Formula weight	911.24	1870.53	983.30
Crystal system	Monoclinic	Triclinic	Orthorhombic
Temperature (K)	120	100	299
Space group	$P2_1/c$	$P\bar{1}$	$Cmc2_1$
θ/°	4.333 to 73.494	2.428 to 76.703	4.2980 to 73.2060
a/Å	16.14702(14)	11.2321(1)	27.3500(3)
b/Å	26.9656(2)	18.6009(2)	11.0237(10)
c/Å	9.27142(9)	21.5863(2)	29.4404(3)
α/Å	90	84.550(1)	90
β/Å	104.8509(10)	89.837(1)	90
γ/Å	90	79.411(1)	90
V/Å	3902.06(6)	4412.63(8)	8876.23(16)
Z	4	2	8
D_c/mg·m^{-3}	1.551	1.408	1.472
μ/mm^{-1}	4.979	4.420	4.427
F (000)	1856.0	1904.0	4000.0
R_1 (I>2σ(I))	0.0247	0.0707	0.0449
wR_2 (all data)	0.0668	0.2002	0.1270
GOF on F^2	1.039	1.061	1.073

Table S2 The proportion of locally excited state (LE) and charge transfer state (CT) of the lowest two singlet (S_1, S_2) computed at ground geometries.

	**1**		**2**		**3**	
	LE (%)	CT (%)	LE (%)	CT (%)	LE (%)	CT (%)
**S_1**	4.991	95.009	2.277	97.723	6.709	93.291
**S_2**	5.035	94.965	2.147	97.853	6.669	93.331

Table S3 Vertical excitation energy levels of the low-lying states (E_{S1}, E_{S2}, E_{T1}, E_{T2}) computed at ground geometries and relaxed excitation energy levels of the low-lying states (E_{S1}, E_{S2}, E_{T1}, E_{T2}) at optimized excited state geometries.

	**vertical excitation energy (eV)**				**relaxed excitation energy (eV)**			
	**E_{S1}**	**E_{S2}**	**E_{T1}**	**E_{T2}**	**E_{S1}**	**E_{S2}**	**E_{T1}**	**E_{T2}**
**1**	3.165	3.172	3.089	3.097	2.964	3.031	2.926	2.970
**2**	2.282	2.283	1.712	1.712	1.895	2.037	1.609	1.644
**3**	2.885	2.885	2.585	2.585	2.596	2.677	2.531	2.571

Table S4 Summary of solid-state luminescent data for compounds **1-3**.

Compounds	λ_{ex} (nm)	$\lambda_{em\text{-}max}$ (nm)	PLQY (%)	τ (ns)	τ_{avg} (ns)
**1**	375	532	9.3	230 (65.19%)<br>3500.84 (34.81%)	1370.61
**2**	375	738	<1	1.24	1.24
**3**	375	553	2.5	12.48 (26.34%)<br>34.75 (73.66%)	28.88

Table S5 The PL lifetimes of compound **1** at different temperatures.

T (K)	τ_1 (ns)	τ_2 (μs)
77	144 (93.10%)	79.06 (6.90%)
97	159 (84.14%)	99.98 (15.86%)
117	108 (61.20%)	137.01 (38.80%)
137	199 (57.29%)	78.08 (42.71%)
157	208 (53.03%)	49.43 (46.97%)
177	229 (51.06%)	33.02 (48.94%)
217	212 (48.39%)	13.44 (51.61%)
237	165 (48.43%)	8.63 (51.57%)
257	134 (50.77%)	5.87 (49.23%)
277	192 (56.01%)	4.21 (43.99%)
297	230 (65.19%)	3.50 (34.81%)

Table S6 The PL lifetimes of compound **2** at different temperatures.

T (K)	τ_1 (ns)	T (K)	τ_1 (ns)
77	4.14	197	3.09
97	3.95	217	2.90
117	3.80	237	2.72
137	3.63	257	2.42
157	3.46	277	1.60
177	3.27	297	1.24

Table S7 The PL lifetimes of compound **3** at different temperatures.

T (K)	τ_1 (ns)	τ_2 (ns)	τ_{avg} (ns)
77	22.40 (32.69%)	55.27 (67.31%)	44.52
97	20.35 (32.47%)	54.17 (67.53%)	43.19
117	19.42 (32.24%)	52.13 (67.76%)	41.58
137	18.30 (30.23%)	50.93 (69.77%)	41.07
157	18.32 (31.70%)	49.60 (68.30%)	39.68
177	16.75 (29.02%)	46.74 (70.98%)	38.04
197	16.29 (26.62%)	44.39 (73.38%)	36.91
217	17.32 (33.65%)	44.19 (66.35%)	35.15
237	13.99 (29.62%)	39.56 (70.38%)	31.98
257	13.05 (29.32%)	38.34 (70.68%)	30.93
277	13.64 (28.28%)	37.24 (71.72%)	30.57
297	12.48 (26.34%)	34.75 (73.66%)	28.88

Table S8 Relationship between dose rate and voltage/current of X-ray source.

Voltage (kV)	Current (μA)	Dose rate (μGy s^{-1})
10	5	0.68844
20	5	4.5830
30	5	9.6410
40	5	11.912
50	5	17.375
50	10	34.750
50	20	69.500
50	30	104.250
50	40	139.000
50	50	173.750
50	60	208.500
50	70	243.250
50	80	278.000

Table S9 Selected bond lengths and angles in compound **1**.

Compound 1			
Cd(1)-O(1)	2.2248(11)	O(4)#1-Cd(1)-O(5)#2	96.10(4)
Cd(1)-O(4)#1	2.2697(11)	O(4)#1-Cd(1)-O(5)	86.48(4)
Cd(1)-O(5)	2.4205(11)	O(4)#1-Cd(1)-N(1)	89.70(5)
Cd(1)-O(5)#2	2.4551(11)	O(4)#1-Cd(1)-N(6)#3	85.96(5)
Cd(1)-N(1)	2.3053(14)	O(5)-Cd(1)-O(5)#2	177.11(4)
Cd(1)-N(6)#3	2.3143(14)	N(1)-Cd(1)-O(5)	94.32(5)
O(1)-Cd(1)-O(4)#1	175.48(4)	N(1)-Cd(1)-O(5)#2	87.03(4)
O(1)-Cd(1)-O(5)	90.13(4)	N(1)-Cd(1)-N(6)#3	173.63(5)
O(1)-Cd(1)-O(5)#2	87.37(4)	N(6)#3-Cd(1)-O(5)	90.08(4)
O(1)-Cd(1)-N(1)	87.57(5)	N(6)#3-Cd(1)-O(5)#2	88.78(4)
O(1)-Cd(1)-N(6)#3	97.04(5)		

Symmetry transformations used to generate equivalent atoms: #1 x-1,-y+3/2,z-1/2 #2 x,-y+3/2,z+1/2 #3 -x,y+1/2,-z+1/2

Table S10 Selected bond lengths and angles in compound **2**.

Compound 2			
Cd(1)-O(1)	2.459(3)	N(1)-Cd(1)-N(12)#1	168.93(14)
Cd(1)-O(2)	2.352(4)	N(12)#1-Cd(1)-O(1)	84.81(13)
Cd(1)-O(5)	2.360(3)	N(12)#1-Cd(1)-O(2)	85.22(14)
Cd(1)-O(6)	2.424(3)	N(12)#1-Cd(1)-O(5)	94.51(14)
Cd(1)-O(11)	2.327(3)	N(12)#1-Cd(1)-O(6)	93.37(13)
Cd(1)-N(1)	2.341(4)	O(3)#2-Cd(2)-O(4)#2	54.54(11)
Cd(1)-N(12)#1	2.350(4)	O(3)#2-Cd(2)-O(7)	157.0(3)
Cd(2)-O(3)#2	2.365(3)	O(12)-Cd(2)-O(3)#2	83.26(13)
Cd(2)-O(4)#2	2.471(3)	O(12)-Cd(2)-O(4)#2	136.93(13)
Cd(2)-O(12)	2.325(4)	O(12)-Cd(2)-O(7)	73.8(3)
Cd(2)-O(7)	2.400(9)	O(12)-Cd(2)-O(8)	128.6(3)
Cd(2)-O(8)	2.338(16)	O(12)-Cd(2)-N(5)	89.88(14)
Cd(2)-N(5)	2.333(4)	O(12)-Cd(2)-N(7)	102.34(14)
Cd(2)-N(7)	2.335(4)	O(7)-Cd(2)-O(4)#2	148.4(3)
O(2)-Cd(1)-O(1)	55.04(12)	O(8)-Cd(2)-O(3)#2	148.1(3)
O(2)-Cd(1)-O(5)	161.90(13)	O(8)-Cd(2)-O(4)#2	93.8(4)
O(2)-Cd(1)-O(6)	143.01(12)	O(8)-Cd(2)-O(7)	54.9(4)
O(5)-Cd(1)-O(1)	143.03(12)	N(5)-Cd(2)-O(3)#2	93.00(13)
O(5)-Cd(1)-O(6)	55.09(12)	N(5)-Cd(2)-O(4)#2	84.73(12)
O(6)-Cd(1)-O(1)	88.00(12)	N(5)-Cd(2)-O(7)	88.8(3)
O(11)-Cd(1)-O(1)	136.23(12)	N(5)-Cd(2)-O(8)	86.4(4)
O(11)-Cd(1)-O(2)	81.39(12)	N(5)-Cd(2)-N(7)	167.77(14)
O(11)-Cd(1)-O(5)	80.51(12)	N(7)-Cd(2)-O(3)#2	88.96(13)
O(11)-Cd(1)-O(6)	135.57(12)	N(7)-Cd(2)-O(4)#2	86.60(12)
O(11)-Cd(1)-N(1)	102.92(14)	N(7)-Cd(2)-O(7)	94.1(3)

O(11)-Cd(1)-N(12)#1	88.01(14)	N(7)-Cd(2)-O(8)	85.6(4)
N(1)-Cd(1)-O(1)	88.40(13)	N(1)-Cd(1)-O(5)	85.68(14)
N(1)-Cd(1)-O(2)	98.02(14)	N(1)-Cd(1)-O(6)	77.64(13)

Symmetry transformations used to generate equivalent atoms: #1 x,y-1,z+1

#2 x,y,z-1

Table S11 Selected bond lengths and angles in compound **3**.

**Compound 3**			
Cd(1)-O(1)	2.430(6)	O(5)-Cd(1)-O(2)	74.1(2)
Cd(1)-O(2)	2.414(6)	O(5)-Cd(1)-O(3)	136.6(2)
Cd(1)-O(3)	2.406(5)	O(5)-Cd(1)-O(4)	83.0(2)
Cd(1)-O(4)	2.424(6)	O(5)-Cd(1)-N(1)	89.06(19)
Cd(1)-O(5)	2.312(4)	O(5)-Cd(1)-N(5)	101.3(2)
Cd(1)-N(1)	2.340(6)	N(1)-Cd(1)-O(1)	90.8(2)
Cd(1)-N(5)	2.324(6)	N(1)-Cd(1)-O(2)	91.0(2)
O(2)-Cd(1)-O(1)	54.32(19)	N(1)-Cd(1)-O(3)	85.2(2)
O(2)-Cd(1)-O(4)	157.08(19)	N(1)-Cd(1)-O(4)	90.7(2)
O(3)-Cd(1)-O(1)	94.67(18)	N(5)-Cd(1)-O(1)	81.9(2)
O(3)-Cd(1)-O(2)	148.76(18)	N(5)-Cd(1)-O(2)	90.7(2)
O(3)-Cd(1)-O(4)	54.13(18)	N(5)-Cd(1)-O(3)	88.0(2)
O(4)-Cd(1)-O(1)	148.50(19)	N(5)-Cd(1)-O(4)	91.8(2)
O(5)-Cd(1)-O(1)	128.4(2)	N(5)-Cd(1)-N(1)	169.6(2)

References

1. Sheldrick, G. M. A Short History of SHELX. Acta Crystallogr., Sect. A: Found. Crystallogr. 2008, 64, 112.

2. Kühne, T. D.; Iannuzzi, M.; Del Ben, M.; Rybkin, V. V.; Seewald, P.; Stein, F.; Laino, T.; Khaliullin, R. Z.; Schütt, O.; Schiffmann, F.; Golze, D.; Wilhelm, J.; Chulkov, S.; Bani-Hashemian, M. H.; Weber, V.; Borštnik, U.; Taillefumier, M.; Jakobovits, A. S.; Lazzaro, A.; Pabst, H.; Müller, T.; Schade, R.; Guidon, M.; Andermatt, S.; Holmberg, N.; Schenter, G. K.; Hehn, A.; Bussy, A.; Belleflamme, F.; Tabacchi, G.; Glöß, A.; Lass, M.; Bethune, I.; Mundy, C. J.; Plessl, C.; Watkins, M.; VandeVondele, J.; Krack, M.; Hutter, J. CP2K: An Electronic Structure and Molecular Dynamics Software Package - Quickstep: Efficient and Accurate Electronic Structure Calculations. *J. Chem. Phys.* 2020, *152* (19), 194103. https://doi.org/10.1063/5.0007045.

3. Perdew, J. P.; Burke, K.; Ernzerhof, M. Generalized Gradient Approximation Made Simple. *Phys. Rev. Lett.* 1996, *77* (18), 3865–3868. https://doi.org/10.1103/PhysRevLett.77.3865.

4. Perdew, J. P.; Burke, K.; Ernzerhof, M. Generalized Gradient Approximation Made Simple [Phys. Rev. Lett. 77, 3865 (1996)]. *Phys. Rev. Lett.* 1997, *78* (7), 1396–1396. https://doi.org/10.1103/PhysRevLett.78.1396.

5. Grimme, S.; Antony, J.; Ehrlich, S.; Krieg, H. A Consistent and Accurate Ab Initio Parametrization of Density Functional Dispersion Correction (DFT-D) for the 94 Elements H-Pu. *J. Chem. Phys.* 2010, *132* (15), 154104. https://doi.org/10.1063/1.3382344.

6. Grimme, S.; Ehrlich, S.; Goerigk, L. Effect of the Damping Function in Dispersion Corrected Density Functional Theory. *J. Comput. Chem.* 2011, *32* (7), 1456–1465. https://doi.org/10.1002/jcc.21759.

7. Adamo, C.; Barone, V. Toward Reliable Density Functional Methods without Adjustable Parameters: The PBE0 Model. *J. Chem. Phys.* 1999, *110* (13), 6158–6170. https://doi.org/10.1063/1.478522.

8. Lippert, G.; Hutter, J.; Parrinello, M. The Gaussian and Augmented-Plane-Wave Density Functional Method for Ab Initio Molecular Dynamics Simulations. *Theor. Chem. Acc.* 1999, *103* (2), 124–140. https://doi.org/10.1007/s002140050523.

9. Lu, T.; Chen, F. Multiwfn: A Multifunctional Wavefunction Analyzer. *J. Comput. Chem.* 2012, *33* (5), 580–592. https://doi.org/10.1002/jcc.22885.

10. Lu, T. A Comprehensive Electron Wavefunction Analysis Toolbox for Chemists, Multiwfn. *J. Chem. Phys.* 2024, *161* (8), 082503. https://doi.org/10.1063/5.0216272.
